# Supplementary material for: Analysing the mechanism of mitochondrial oxidation-induced cell death using a multifunctional iridium(III) photosensitiser
Source: Nat Commun. 2021 Jan 4;12:26. doi: 10.1038/s41467-020-20210-3 (PMC7782791; doi:10.1038/s41467-020-20210-3)
Supplement: Supplementary file 1 — Supplementary Information [file 41467_2020_20210_MOESM1_ESM.pdf]

Supplementary Information

Analysing the Mechanism of Mitochondrial Oxidation-induced Cell Death Using a Multifunctional Iridium(III) Photosensitiser

Chaiheon Lee[†], Jung Seung Nam[†], Chae Gyu Lee, Mingyu Park, Chang-Mo Yoo, Hyun-Woo Rhee, Jeong Kon Seo, and Tae-Hyuk Kwon**

C. Lee,[†] J. S. Nam,[†] C. G. Lee, M. Park, Prof. Dr. T.-H. Kwon

Department of Chemistry and Center for Wave Energy Materials, Natural Science Building, Ulsan National Institute of Science and Technology (UNIST), Ulsan 44919 (Republic of Korea)

E-mail: kwon90@unist.ac.kr

Prof. Dr. J. K. Seo

UNIST Central Research Facility (UCRF), Ulsan National Institute of Science and Technology (UNIST), Ulsan 44919 (Republic of Korea)

E-mail: jkseo6998@unist.ac.kr

C. Yoo, Prof. Dr. H.-W. Rhee

Department of Chemistry, Seoul National University, Seoul 08826 (Republic of Korea)

E-mail: rheehw@snu.ac.kr

*To whom correspondence should be addressed: jkseo6998@unist.ac.kr, kwon90@unist.ac.kr

Table of Contents

Supplementary Method 1. Materials.

Supplementary Method 2. Synthesis and analysis of Ir-OA and Ir-OC.

Supplementary Method 3. Photophysical properties analysis (Absorbance and Photoluminescence).

Supplementary Method 4. ROS generation assay: ABDA assay ($^1O_2$), DHR123 assay ($O_2^{\cdot -}$).

Supplementary Method 5. Cell culturing, subcellular localisation imaging.

Supplementary Method 6. Intracellular ROS generation assay: H_2DCF-DA assay.

Supplementary Method 7. Cell viability test (live or dead assay, MTT assay, and CCK-8 assay).

Supplementary Method 8. Time correlated single photon counting (TCSPC).

Supplementary Method 9. Mitochondrial viscosity monitoring.

Supplementary Method 10. Mitochondrial depolarisation monitoring.

Supplementary Method 11. Western blot for protein photo-crosslinking.

Supplementary Method 12. Line-cut analysis of western blot for cross-linking and calculation of correlation value.

Supplementary Method 13. Mitochondrial membrane potential assay (TMRE assay).

Supplementary Method 14. Mitochondrial morphology monitoring.

Supplementary Figure 1-9. ^{1}H NMR spectra and ^{13}C NMR spectra.

Supplementary Figure 10. Absorption and emission spectra of energy donor and acceptor.

Supplementary Figure 11. H_2DCF-DA assay for identification of ROS generation assay inside live cells.

Supplementary Figure 12. Localisation of **Ir-OC** in the living cells.

Supplementary Figure 13. MTT-assay for HeLa with **Ir-OA** and **Ir-OC.**

Supplementary Figure 14. Real-time imaging for in vitro photodynamic therapy.

Supplementary Figure 15. Representative flow cytometry plot for HeLa cells without iridium complexes.

Supplementary Figure 16. Lifetime analysis for **Ir-OA** depending on viscosity.

Supplementary Figure 17. The change in the lifetime of **Ir-OA** according to BSA concentration.

Supplementary Figure 18. Ponceau S staining for identifying protein loading quantity.

Supplementary Figure 19. Protein photo-crosslinking by photoactivation of **Ir-OA** in HeLa cells

Supplementary Figure 20. Intermolecular energy transfer efficiency depending on solvent polarity.

Supplementary Figure 21. Wavelength resolved CLSM images of mitochondrial depolarisation.

Supplementary Figure 22. TMRE assay for monitoring mitochondria depolarisation.

Supplementary Table 1. Photophysical properties of **Compound 4, Ir-OA** and **Ir-OC.**

Supplementary Table 2. Quantitative phototoxicity of **Ir-OA** according to irradiation energy.

Supplementary Table 3. Detail information of prepared plasmids

Supplementary Method 1. Materials and methods. Utilised chemical reagents for synthesis and analysis were purchased adequately from commercial suppliers (Sigma Aldrich, Alfa Aesar, Acros organics, Tokyo Chemical Industry, Strem Chemicals, JUNSEI, FMC corporation and SAMCUHUN). All the solvents were purchased from SAMCHUN Chemicals, Republic of Korea and information of suppliers were denoted if it was considered a necessity. $[Ru(bpy)_3]Cl_2$ (Sigma Aldrich, USA) was used for comparative analysis with **Ir-OA** and **Ir-OC**. All synthesised compounds and complexes were analysed by ^1H, ^{13}C NMR (Agilent 400MR-DD2 NMR spectroscopy), FT-IR (Varian Cary 620/670 FT-IR spectrometer, UNIST Central Research Facilities, Ulsan, Republic of Korea) and HR-MS (Bruker maXis™ HD Ultra-high resolution Q-TOF LC-MS/MS system, The Cooperative Laboratory Center of Pukyong National University, Republic of Korea), respectively. Photophysical studies for each complex were proceeded by an UV-visible spectrometer (SHIMADZU UV-2600 240V EN, Japan) and fluorescence spectrometer (ISS PC1 photon counting spectrofluorometer, USA). HeLa cells were purchased from Korean cell line bank, Republic of Korea. Cell images for HeLa cells and HEK293T cells were obtained on Carl Zeiss LSM780NLO and LSM980 confocal laser scanning microscope, Germany. Materials for reactive oxygen species analyses, 9,10-anthracenediyl-bis(methylene)dimalonic acid (ABDA) (Sigma Aldrich, USA) were used for singlet oxygen assay, Dihydrorhodamine123 (Cayman chemical company, USA) were employed for superoxide radical anion assay, and 2′,7′-Dichlorodihydrofluorescein diacetate (H_2DCF-DA) (Sigma Aldrich, USA) were purchased for ROS generation assay inside live cells. Bovine serum albumin (BSA) (Sigma Aldrich, USA) was utilised as reference proteins for phosphorescence lifetime study Materials for mitochondrial MMP analysis, Tetramethylrhodamine Ethyl Ester Perchlorate (TMRE) were purchased from Invitrogen, USA. For cell viability test, 3-(4,5-dimethyl-2-thiazolyl)-2,5-diphenyl-2H-tetrazolium bromide (MTT) (Alfa Aesar, USA) and CCK-8 (Abcam, UK) were used. The flow cytometric analysis was experimented on BD FACSVerse™ (BD bioscience, US). All plots and graphs are processed by OriginPro 2017 and Microsoft office 2017.

Supplementary Method 2. Synthesis and analysis of Ir-OA and Ir-OC.

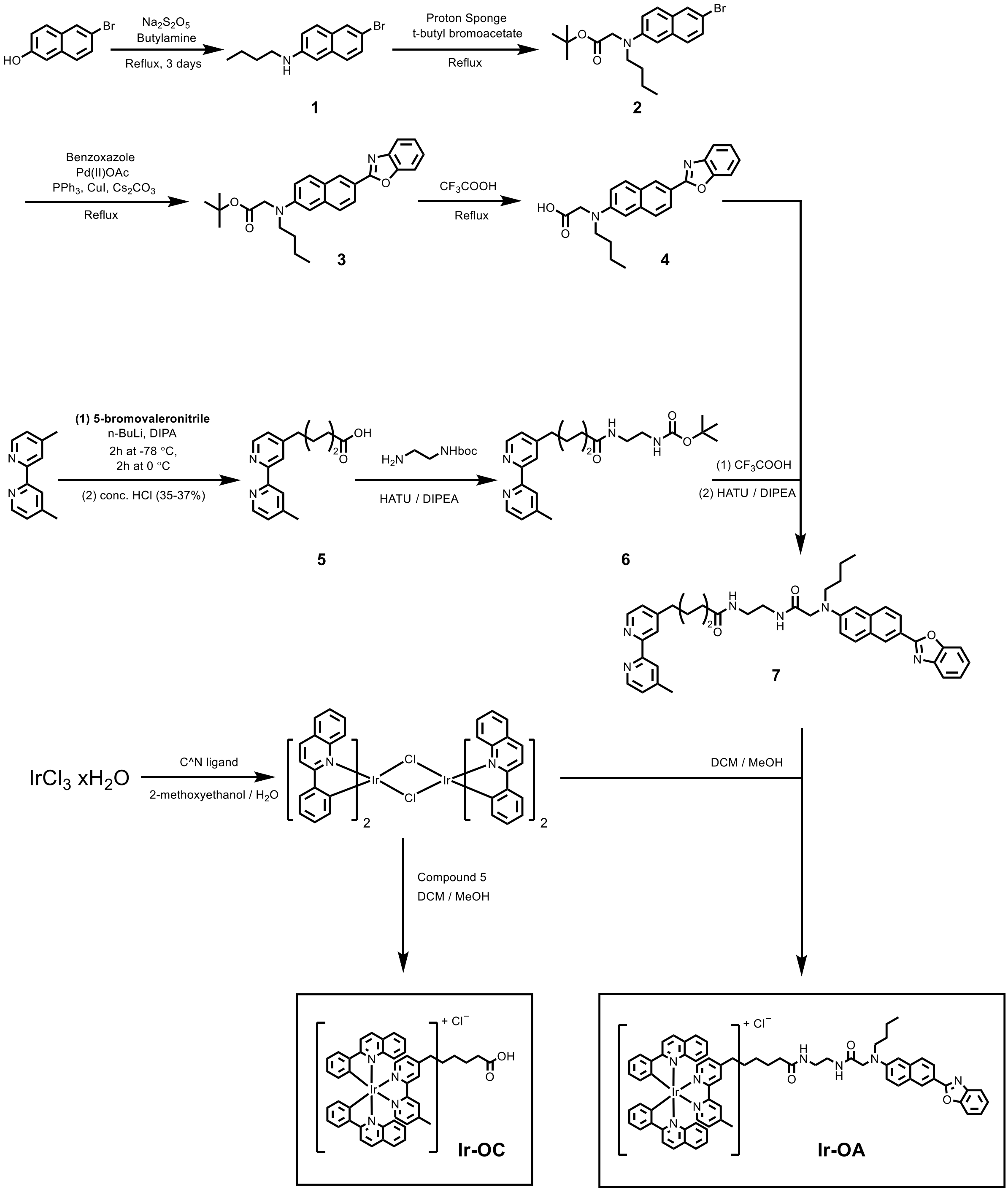

Scheme S1. Synthetic pathway of **Ir-OA** and **Ir-OC**.

(1) Synthesis of 6-bromo-*N*-butylnaphthalen-2-amine (Compound 1). A round bottom flask was charged with 6-Bromo-2-naphthol (3.00 g, 13.5 mmol), $Na_2S_2O_5$ (3.07 g, 16.1 mmol), 1-butylamine (3.34 mL, 33.8 mmol), and DI water (100 mL). The solution was refluxed for 48 hours and cooled down to room temperature. Then, the reaction mixture was poured into 2N NaOH aqueous solution. After stirring for 1 hour at 0 °C, the precipitated product was filtered and washed with cold DI water. Yield: 67%. ^1H NMR (400 MHz, d_6-DMSO): δ (ppm) = 7.851 (d, J = 2.0 Hz, 1H), 7.563 (d, J = 8.8 Hz, 1H), 7.524 (d, J = 9.2 Hz, 1H), 7.362 (dd, J = 8.8 Hz, 2.0 Hz, 1H), 7.007 (dd, J = 8.8 Hz, 2.4 Hz, 1H), 6.680 (d, J = 2.4 Hz, 1H), 6.020 (t, J = 5.2 Hz, 1H), 3.077 (td, J = 6.8 Hz, 5.2 Hz, 2H), 1.594 (m, 2H), 1.420 (m, 2H), 0.932 (t, J = 7.6 Hz, 3H), ^{13}C NMR (100 MHz, chloroform-d): δ (ppm) = 146.30, 133.74, 129.53, 129.38, 128.36, 127.93, 127.50, 118.84, 114.83, 103.96, 43.64, 31.40, 20.33, 13.91. HR-MS: m/z calculated for $C_{14}H_{16}BrN$: 278.0539 ($[M+H]^+$), found: 278.0538 ($[M+H]^+$). FT-IR (neat cm^{-1}): 653,

734, 765, 808, 871, 890, 914, 956, 1062, 1076, 1143, 1168, 1216, 1294, 1375, 1400, 1461, 1511, 1589, 1627, 2823, 2869, 2925, 2948, 3355 (br).

(2) Synthesis of *tert*-butyl *N*-(6-bromonaphthalen-2-yl)-*N*-butylglycinate (Compound 2). A solution of compound 1 (1.24 g, 4.48 mmol), proton sponge (1.44 g, 6.71 mmol), tert-butyl bromoacetate (3.16 g, 16.12 mmol) in MeCN (50 mL) was refluxed under inert condition. After 24 hours, the reaction mixture was cooled down to room temperature, and the product was extracted three times with ethyl acetate, washed with brine. The extracted product was purified by silica gel column chromatography (elution with hexane/ethyl acetate v/v = 6:1) as yellow oil. Yield: 86%. ^{1}H NMR (400 MHz, d_6-DMSO): δ (ppm) = 7.925 (d, *J* = 2.0 Hz, 1H), 7.696 (d, *J* = 9.2 Hz, 1H), 7.575 (d, *J* = 8.8 Hz, 1H), 7.404 (dd, *J* = 9.2 Hz, 2.0 Hz, 1H), 7.100 (dd, *J* = 9.2 Hz, 2.4 Hz, 1H), 6.794 (d, *J* = 2.8 Hz, 1H), 4.130 (s, 2H), 3.423 (t, *J* = 7.6 Hz, 2H), 1.575 (m, 2H), 1.390 (s, 9H), 1.370 (m, 2H), 0.921 (t, *J* = 7.6 Hz, 3H), ^{13}C NMR (100 MHz, chloroform-d): δ (ppm) = 170.22, 146.34, 133.46, 129.31, 129.28, 127.97, 127.77, 127.73, 116.19, 114.93, 105.58, 81.66, 53.88, 52.12, 29.84, 28.04 (3C), 20.30, 14.01, HR-MS: m/z calculated for $C_{20}H_{26}BrNO_2$: 392.1220 ([M+H]$^+$), found: 392,1217 ([M+H]$^+$). FT-IR (neat, cm^{-1}): 746, 798, 840, 875, 937, 1060, 1126, 1153, 1209, 1253, 1274,1292, 1367, 1394, 1434, 1456, 1506, 1589, 1623, 1743, 2871, 2931, 2958.

(3) Synthesis of *tert*-butyl *N*-(6-(benzo[d]oxazol-2-yl)naphthalen-2-yl)-*N*-butylglycinate (Compound 3). A solution of compound 2 (621 mg, 1.59 mmol), benzoxazole (226 mg, 1.90 mmol), Pd(II)$(OAc)_2$ (54 mg, 0.08 mmol), PPh3 (42 mg, 0.16 mmol), CuI (60 mg, 0.32 mmol), and Cs_2CO_3 (620 mg, 1.90 mmol) in degassed DMF (80 mL) was refluxed at 140°C under Ar. After 16 hours, the reaction mixture was cooled down to room temperature, and filtered. The product was extracted three times with ethyl acetate and washed three times with brine. The crude product was purified by silica gel column chromatography (elution with (i) hexane/ethyl acetate v/v = 5:1, then (ii) hexane/ethyl acetate v/v = 7:1) as pale yellow solid. Yield: 52%. ^{1}H NMR (400 MHz, d_6-DMSO): δ (ppm) = 8.595 (d, *J* = 1.6 Hz, 1H), 8.072 (dd, *J* = 8.8 Hz, 1.6 Hz, 1H), 7.957 (d, *J* = 9.2 Hz, 1H), 7.779 (m, 3H), 7.043 (m, 2H), 7.168 (dd, *J* = 9.2 Hz, 2.8 Hz, 1H), 6.874 (d, *J* = 2.4 Hz, 1H), 4.204 (s, 2H), 3.485 (t, *J* = 8.0 Hz, 2H), 1.611 (p, *J* = 8.4 Hz, 2H), 1.416 (s, 9H), 1.387 (m, 2H), 0.941 (t, *J* = 7.2 Hz, 3H), ^{13}C NMR (100 MHz, chloroform-d): δ (ppm) = 169.98, 163.96, 150.71, 147.64, 142.30, 136.73, 130.20, 128.03, 126.70, 125.93, 124.57, 124.40, 120.02, 119.55, 115.94, 110.35, 105.41, 81.84, 53.84, 52.18, 29.79, 28.06 (3C), 20.29, 14.01, HR-MS: m/z calculated for $C_{27}H_{30}N_2O_3$: 431.2329 ([M+H]$^+$), found: 431.2330 ([M+H]$^+$). FT-IR (neat, cm^{-1}): 659, 740, 782, 802, 848, 894, 933, 950, 1002, 1049, 1106, 1135, 1160, 1211, 1245, 1276, 1294, 1351, 1367, 1390, 1421, 1488, 1506, 1550, 1602, 1625, 1724, 1737, 2856, 2927, 2054.

(4) Synthesis of *N*-(6-(benzo[d]oxazol-2-yl)naphthalen-2-yl)-*N*-butylglycine (Compound 4). A solution of compound 3 (400 mg, 0.93 mmol), trifluoroacetic acid (1.5 mL), in DCM (10 mL) was stirred at room temperature under Ar. After 16 hours, the solvent was removed under vacuum condition. The crude product was purified by trituration with chloroform and hexane. The product was washed by hexane and obtained as pale-yellow powder. Yield: 94%. ^{1}H NMR (400 MHz, d_6-DMSO): δ (ppm) = 12.682 (s, 1H), 8.589 (s, 1H), 8.063 (d, *J* = 8.4 Hz, 1H), 7.944 (d, *J* = 2.8 Hz, 1H), 7.778 (m, 3H), 7.401 (m, 2H), 7.180 (dd, *J* = 8.0 Hz, 1.6 Hz, 1H), 6.907 (s, 1H), 4.233 (s, 2H), 3.486 (t, *J* = 7.2 Hz, 2H), 1.619 (p, 7.2 Hz, 2H), 1.383 (m, 2H), 0.946 (t, *J* = 7.2 Hz, 3H), ^{13}C NMR (100 MHz, chloroform-d): δ (ppm) = 170.16, 161.53, 148.63, 146.04, 140.36, 134.75, 128.26, 125.91, 124.85, 123.55, 122.94, 122.69, 122.28, 117.59, 117.57, 114.33, 108.66, 102.95, 50.44, 49.77, 27.64, 18.16, 12.25. HR-MS: m/z calculated for $C_{23}H_{22}N_2O_3$: 375.1703 ([M+H]$^+$), found: 375.1705 ([M+H]$^+$). FT-IR (neat, cm^{-1}): 725, 742, 798, 842, 873, 892, 950, 1004, 1058, 1108, 1133, 1197, 1213, 1247, 1278, 1311, 1363, 1390, 1403, 1456, 1506, 1546, 1625, 1720, 2867, 2902, 2933, 2954. 2700-2900 (br).

(5) Synthesis of 6-(4'-methyl-[2,2'-bipyridin]-4-yl)hexanoic acid (Compound 5). The compound 5 was prepared by literature methods[1], Yield: 77%. ^{1}H NMR (400 MHz, d_6-DMSO): δ (ppm) = 12.014 (s, 1H), 8.524 (t, *J* = 4.8 Hz, 2H), 8.223 (s, 2H), 7.262 (m, 2H), 2.656 (t, *J* = 8.0 Hz, 2H), 2.393 (s, 3H), 2.192 (t, *J* = 7.6 Hz, 2H), 1.611 (p, *J* = 7.6 Hz, 2H), 1.527 (p, *J* = 7.6 Hz, 2H), 1.298 (p, *J* = 7.2 Hz, 2H), ^{13}C NMR (100 MHz, chloroform-d, d_6-DMSO): δ (ppm) = 177.76, 155.58, 155.40, 152.80, 148.95, 148.89, 148.41, 124.83, 124.14, 122.67, 121.83, 35.04, 34.01, 29.74, 28.29, 24.50, 21.24. HR-MS: m/z calculated for $C_{17}H_{20}N_2O_2$: 285.1598 ([M+H]$^+$), found: 285.1602 ([M+H]$^+$). FT-IR (neat, cm^{-1}): 674, 723, 761, 827, 856, 898, 927, 1006, 1039, 1079, 1116, 1133, 1180, 1209, 1224, 1245, 1276, 1295, 1344, 1413, 1425, 1467, 1550, 1598, 1708, 2863, 2942, 2400-2600 (br), 2800-3000 (br)

(6) Synthesis of *tert*-butyl (2-(6-(4'-methyl-[2,2'-bipyridin]-4-yl)hexanamido) ethyl)carbamate (Compound 6). A solution of compound 5 (300 mg, 1.06 mmol), O-(7-Azabenzotirazol-1-yl)-*N,N,N',N'*-etramethyluronium hexafluorophosphate (HATU) (361 mg, 0.95 mmol), and *N,N*-diisopropylethylamine (409 mg, 3.17 mmol) in degassed DMF was stirred for 15 min. To the solution was added *N*-boc-ethylenediamine (253 mg, 1.59 mmol)

slowly at the room temperature. After 5 hours, the product was extracted with ethyl acetate and purified by silica gel column chromatography (elution with (i) chloroform/methanol v/v = 10:1) as white powder. Yield: 89%. ^{1}H NMR (400 MHz, d_6-DMSO): δ (ppm) = 8.531 (t, *J* = 5.6 Hz, 2H), 8.223 (s, 2H), 7.773 (t, *J* = 5.6 Hz, 2H), 7.276 (m, 2H), 6.767 (t, *J* = 5.6 Hz, 2H), 3.034 (q, *J* = 6.0 Hz, 2H), 2.937 (q, *J* = 6.0 Hz, 2H), 2.674 (t, *J* = 8.0 Hz, 2H), 2.408 (s, 3H), 2.038 (t, *J* = 7.6 Hz, 2H), 1.623 (p, *J* = 7.6 Hz, 2H), 1.528 (p, 7.6 Hz, 2H), 1.362 (s, 9H), 1.281 (p, *J* = 7.6 Hz, 2H), ^{13}C NMR (100 MHz, chloroform-d): δ (ppm) = 173.55, 155.87, 152.57, 148.99, 148.69, 148.64, 148.40, 124.68, 124.68, 123.99, 122.14, 121.29, 79.65, 40.79, 40.25, 36.46, 35.24, 29.95, 28.80, 28.34 (3C), 25.32, 21.20. HR-MS: m/z calculated for $C_{24}H_{34}N_4O_3$: 427.2704 ([M+H]$^+$), found: 427.2710 ([M+H]$^+$). FT-IR (neat, cm^{-1}): 671, 686, 709, 769, 784, 821, 852, 867, 900, 973, 991, 1039, 1054, 1168, 1205, 1251, 1282, 1317, 1342, 1369, 1419, 1446, 1461, 1538, 1596, 1650, 1679, 2863, 2940, 2987, 3324, 3351.

(7) Synthesis of *N*-(2-(2-((6-(benzo[d]oxazol-2-yl)naphthalen-2-yl)(butyl)amino) acetamido)ethyl)-6-(4'-methyl-[2,2'-bipyridin]-4-yl)hexanamide (Compound 7). To a solution of compound 6 (100 mg, 0.23 mmol) in DCM (10 mL) was added trifluoroacetic acid (4 mL) slowly for deprotection of Boc. After stirring for 2 hours, the deprotected compound 6 was obtained by removing solvent with rotary evaporator. A solution of the compound 4 (88 mg, 0.234 mmol), *O*-(7-Azabenzotirazol-1-yl)-*N,N,N',N'*-etramethyluronium hexafluorophosphate (HATU) (80 mg, 0.21 mmol), and N,N-diisopropylethylamine (91 mg, 0.70 mmol) in degassed DMF (10 mL) was stirred for 15 min. To the solution was added the deprotected compound 6 slowly at the room temperature. After 5 hours, the product was extracted with ethyl acetate and purified by silica gel column chromatography (elution with (i) DCM/methanol v/v = 20:1) as pale-yellow powder. Yield: 49%. ^{1}H NMR (400 MHz, d_6-DMSO): δ (ppm) = 8.579 (d, *J* = 1.2 Hz, 1H), 8.511 (d, *J* = 3.2 Hz, 1H), 8.499 (d, *J* = 3.2 Hz, 1H), 8.198 (d, *J* = 6.8 Hz, 2H), 8.055 (dd, *J* = 8.8 Hz, 1.6 Hz, 1H), 7.968 (d, *J* = 4.8 Hz, 1H), 7.942 (d, *J* = 9.3 Hz, 1H), 7.786 (d, *J* = 8.4 Hz, 1H), 7.754 (m, 3H), 7.391 (m, 2H), 7.256 (dq, *J* = 4.8 Hz, 0.8 Hz, 1H), 7.217 (dd, *J* = 5.2, 1.6 Hz, 1H), 7.152 (dd, *J* = 9.2 Hz, 2.4 Hz, 1H), 6.902 (d, *J* = 2.4 Hz, 1H), 4.008 (s, 2H), 3.488 (t, *J* = 7.6 Hz, 2H), 3.110 (m, 4H), 2.611 (t, *J* = 7.6 Hz, 2H), 2.398 (s, 3H), 1.947 (t, *J* = 7.6 Hz, 2H), 1.574 (m, 4H), 1.451 (p, *J* = 7.6 Hz, 2H), 1.360 (m, 2H), 1.210 (m, 2H), 0.929 (t, *J* = 7.6 Hz, 2H), ^{13}C NMR (100 MHz, chloroform-d): δ (ppm) = 173.87, 170.88, 163.53, 155.85, 155.23, 152.44, 150.73, 148.96, 148.69, 148.36, 146.94, 142.30, 136.39, 130.43, 127.82, 126.85, 126.37, 124.82, 124.79, 124.67, 124.50, 123.90, 122.12, 121.14, 121.04, 119.70, 116.26, 110.41, 106.46, 55.77, 52.13, 40.29, 39.23, 35.97, 35.09, 29.72, 29.04, 28.67, 25.06, 21.20, 20.24, 13.93. HR-MS: m/z calculated for $C_{42}H_{46}N_6O_3$: 683.3704 ([M+H]$^+$), found: 683.3705 ([M+H]$^+$). FT-IR (neat, cm^{-1}): 667, 678, 696, 740, 759, 782, 802, 825, 844, 898, 946, 1000, 1049, 1108, 1130, 1184, 1213, 1245, 1373, 1403, 1454, 1504, 1548, 1596, 1627, 1644, 2861, 2933, 3299.

(8) Synthesis of Ir-OC. A solution of **compound 5** (30 mg, 0.02 mmol) and $(2pq)_4Ir_2Cl_2$ (14 mg, 0.05 mmol) (prepared by literature methods[2]) in DCM/MeOH (10 mL, v/v = 1:1) was refluxed under Ar condition for 16 hours. The solvent was removed by rotary evaporator. The crude was dissolved in 20 mL EtOH and filtered using Büchner funnel to remove $(2pq)_4Ir_2Cl_2$ which is not soluble to EtOH. The filtrate was evaporated again, and the products was triturated with DCM and hexane to obtain orange powder. Yield: 97%. ^{1}H NMR (400 MHz, d_6-DMSO): δ (ppm) = 12.015 (s, 1H), 8.548 (m, 4H), 8.358 (s, 1H), 8.320 (s, 1H), 8.281 (d, *J* = 8.0 Hz, 2H), 7.922 (m, 4H), 7.526 (dd, *J* = 6.4, 1.6 Hz, 1H), 7.501 (d, *J* = 6.0 Hz, 1 H), 7.423 (t, *J* = 7.2 Hz, 2H), 7.242 (d, *J* = 9.2 Hz, 1H), 7.206 (d, *J* = 9.2 Hz, 1H), 7.145 (t, *J* = 6.8 Hz, 2H), 7.059 (m, 2H), 6.807 (t, *J* = 7.6 Hz, 2H), 6.403 (t, *J* = 7.2 Hz, 2H), 2.645 (t, *J* = 7.6 Hz, 2H), 2.397 (s, 3H), 2.156 (t, *J* = 7.2 Hz, 2H), 1.538 (p, *J* = 7.6 Hz, 2H), 1.458 (p, *J* = 7.6 Hz, 2H), 1.134 (m, 2H), ^{13}C NMR (100 MHz, chloroform-d): δ (ppm) = 175.87, 169.87, 169.79, 156.14, 155.72, 155.44, 155.42, 152.22, 151.55, 151.45, 147.52, 147.51, 146.65, 146.32, 146.30, 145.56, 145.54, 139.82, 139.77, 134.65, 134.61, 131.14, 130.75, 130.73, 128.95, 128.09, 127.53, 127.49, 127.03, 126.85, 126.83, 126.77, 126.22, 126.20, 124.96, 124.78, 124.75, 122.75, 122.71, 117.30, 117.16, 34.62, 34.30, 28.77, 27.39, 24.05, 21.29. HR-MS: m/z calculated for $C_{47}H_{40}IrN_8O_3$: 885.2784 (M$^+$), found: 885.2780 (M$^+$). FT-IR (neat, cm^{-1}): 655, 682, 711, 727, 738, 765, 788, 835, 1039, 1072, 1147, 1166, 1216, 1243, 1276, 1292, 1309, 1338, 1378, 1425, 1448, 1463, 1484, 1515, 1546, 1581, 1606, 1724, 2044, 2561, 2856, 2937, 3048, 2400-2700 (br), 3100-3500 (br). HPLC purity: 95.8%. Elemental analysis: calculated for $C_{47}H_{40}IrN_4O_2Cl$ $1.6H_2O$ = C:59.46, H:4.59, N:5.90, O, 5.91, found: C:58.72, H:4.43, N:5.78, O:5.98

(9) Synthesis of Ir-OA. A solution of **compound 7** (400 mg, 0.59 mmol) and $(2pq)_4Ir_2Cl_2$ (373 mg, 0.29 mmol) DCM/MeOH (10 mL, v/v = 1:1) was refluxed under Ar condition for 16 hours. The solvent was removed by rotary evaporator. Next procedure is the same as above **Ir-OC.** The product was obtained as an orange powder. Yield: 88%. ^{1}H NMR (400 MHz, d_6-DMSO): δ (ppm) = 8.535 (m, 5H), 8.342 (s, 1H), 8.266 (m, 3H), 8.046 (t, *J* = 5.2 Hz, 1H), 8.016 (dd, *J* = 8.4 Hz, 2.0 Hz, 1H), 7.907 (m, 5H), 7.790 (t, 5.2 Hz, 1H), 7.746 (d, 8.8 Hz, 1H), 7.726 (m, 2H), 7.482 (d, *J* = 5.6 Hz, 1H), 7.404 (m, 5H), 7.239 (d, *J* = 8.8 Hz, 1H), 7.179 (d, *J* = 8.8 Hz, 1H), 7.139 (m,

3H), 7.081 (t, J = 8.0 Hz, 1H), 7.003 (t, J = 8.0 Hz, 1H), 6.888 (d, J = 2.0 Hz, 1H), 6.801 (dd, J = 7.6, 2.8 Hz, 2H), 6.399 (dd, J = 7.6, 2.4 Hz, 2H), 4.015 (s, 2H), 3.476 (t, J = 7.6 Hz, 2H), 3.107 (m, 4H), 2.564 (t, J = 7.6 Hz, 2H), 2.368 (s, 3H), 1.915 (t, J = 7.6 Hz, 2H), 1.585 (p, 7.2 Hz, 2H), 1.449 (p, J = 7.2 Hz, 2H), 1.349 (m, 4H), 1.036 (m, 2H), 0.897 (t, J = 7.6 Hz, 3H), ^{13}C NMR (100 MHz, chloroform-d): δ (ppm) = 174.84, 170.21, 169.88, 169.82, 164.08, 156.44, 155.72, 155.58, 152.12, 151.66, 151.56, 150.65, 147.83, 147.54, 147.46, 146.44, 146.15, 145.50, 145.45, 142.41, 139.66, 139.63, 136.76, 134.62, 131.04, 131.00, 130.76, 130.74, 129.93, 128.86, 128.83, 127.90, 127.83, 127.45, 127.42, 127.41, 126.90, 126.85, 126.77, 126.75, 126.71, 126.47, 125.73, 125.19, 124.78, 124.73, 124.46, 124.32, 123.91, 122.71, 122.67, 119.51, 119.44, 117.18, 117.06, 116.46, 110.28, 105.53, 77.20, 54.79, 51.68, 40.73, 38.69, 34.72, 34.62, 28.96, 28.36, 27.25, 24.23, 21.03, 20.20, 14.00. HR-MS: m/z calculated for $C_{72}H_{66}IrN_8O_3$: 1283.4894 (M^+), found: 1283.4888 (M^+). FT-IR (neat, cm^{-1}): 680, 700, 727, 750, 765, 802, 833, 900, 948, 1002, 1039, 1070, 1130, 1147, 1180, 1197, 1214, 1243, 1290, 1340, 1367, 1425, 1450, 1515, 1546, 1579, 1619, 1658, 2871, 2931, 3060, 3100-3500 (br), HPLC purity: 96.5%. Elemental analysis: calculated for $C_{72}H_{66}IrN_8O_3Cl$ 5.7H_2O = C:60.83, H:5.49, N:7.88, O:9.79, found: C:60:22, H:5.24, N:7.83, O:9.81.

Supplementary Method 3. Photophysical properties analysis (Absorbance and Photoluminescence). The N_2 bubbled 20 μM solutions of **Compound 4**, **Ir-OC**, and **Ir-OA** in H_2O:DMSO = 99:1 (v/v%) condition was prepared for analysing photophysical properties with UV-visible spectrometer (SHIMADZU UV-2600 240V EN, Japan) and fluorescence spectrometer (ISS PC1 photon counting spectrofluorometer, USA). A 1 cm × 1 cm cuvette cell (Hellma) and 1 cm × 0.2 cm cuvette cell (Hellma) was used to measure absorbance and photoluminescence respectively. The energy transfer efficiency (η_{ET}) was measured from the steady-state photoluminescence spectra as $\eta_{ET} = 1-(A_{D-A}/A_D)$, where A_{D-A} and A_D are the integral area (emission range of donor: 400-520 nm) of **Ir-OA** and compound 4. Following quantum yield for photoluminescence (Φ_{PL}) was calculated with equation: $\Phi_{PL} = \Phi_{ref} \times (A_{ref}/A) \times (I/I_{ref})$. The $[Ru(bpy)_3]^{2+}$ was used as reference that was previously reported (Table S1)[3] where A is absorbance, and I is integrated emission intensity (λ_{ex} = 400 nm)

To verify the interaction between iridium ligands and energy donor, we measured the emission spectrum of compound 3 with or without $[(2pq)_2Ir(bpy)]^+Cl^-$, iridium complex without energy donor, in MeOH and water. In the MeOH, the compound 3 with $[(2pq)_2Ir(bpy)]^+Cl^-$ showed similar emission intensity to the case without $[(2pq)_2Ir(bpy)]^+Cl^-$. In the water, however, the emission intensity of compound 3 dramatically decreased when the $[(2pq)_2Ir(bpy)]^+Cl^-$ is additionally dissolved, which implies that intermolecular energy transfer efficiency increased in hydrophilic environment due to strengthen π-π interaction between energy donor and iridium ligands (Figure S15).

The polarity dependent emission property of **Ir-OA** was measured by the solution of fluorescence spectrometer (ISS PC1 photon counting spectrofluorometer, USA). Eleven solutions of 20 μM **Ir-OA** in H_2O:MeOH (0:10, 1:9, 2:8, … ,10:0, volume ratio) solvent were prepared.

Supplementary Method 4. ROS generation assay: ABDA assay ($^1O_2$), DHR123 assay ($O_2^{\cdot-}$). The singlet oxygen ($^1O_2$) generation was measured by $^1O_2$ indicator, 9,10-anthracenediyl-bi(methylene)dimalonic acid (ABDA). The photo-oxidation of ABDA with $^1O_2$ forms peroxide bridge on an anthracene core, which degrades its absorbance at 384 nm. To each 4 μM solution of Compound 4, **Ir-OC**, **Ir-OA**, and $[Ru(bpy)_3]^{2+}$ was added 100 mM stock solution of ABDA to 1000:1 volume ratio (1:25 molar ratio). These solutions were irradiated by LED array (λ = 400 nm, 1.42 mW cm^{-2}) for 0, 2, 4, 6, 8, and 10 min, and the ABDA absorbance at 384 nm was measured by microplatereader. The ABDA absorbance decay of each sample was recorded as $Abs_{ABDA,\ 384\ nm}$ - $Abs_{sample,\ 384\ nm}$ to correct baseline. The superoxide radical anion ($O_2^{\cdot-}$) generation was measured by $O_2^{\cdot-}$ indicator, Dihydrorhodamine123 (DHR123). The DHR123 is oxidised by $O_2^{\cdot-}$ to form rhordamine123 which emit strong

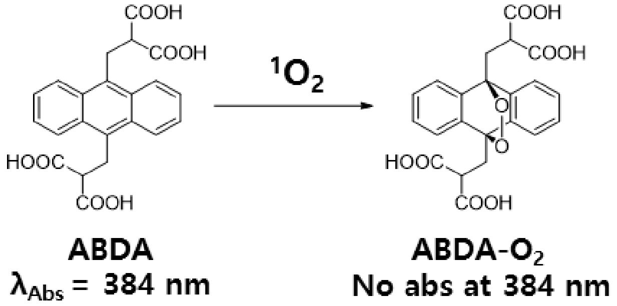

green fluorescence. To each 4 μM solution of Compound 4, **Ir-OC**, **Ir-OA**, and $[Ru(bpy)_3]^{2+}$ was added 4 mM and dihydrorhodamine123 stock solution in DMSO as 1000:1 volume ratio (1:1 molar ratio). Each solution was irradiated by LED array (λ = 400 nm, 1.42 mW cm^{-2}) for 0, 2, 4, 6, 8, and 10 min, and the green fluorescence from rhodamine123 was recorded for each sample with microplatereader ($\lambda_{excitation}$ = 507 nm, $\lambda_{emission}$ = 529 nm).

Supplementary Method 5. Cell culturing, subcellular localisation imaging. HEK293T and Hela cells were cultured on the cell culture plate in the DMEM (Gibco) supplemented with 10% FBS, 50 units/mL penicillin, and 50 μg/mL streptomycin. The cells were grown at 37 °C under humidified atmosphere and 5% CO_2. For the subcellular localisation imaging with confocal laser scanning microscopy (CLSM), the HeLa cells were grown 24 hours before imaging on the coverglass-bottom confocal dish (coverglass size: 22x22x0.13 mm) and incubated with culture media of 4 μM **Ir-OC** and **Ir-OA** for 90 min (Culture media:DMSO = 1000:1, v/v). Then, the HeLa cells were washed several times with DPBS and incubated with culture media of 100 nM MitoTracker™ Deep Red FM (Invitrogen, M22426). After washing several times with DPBS, the HeLa cells were imaged with Carl Zeiss LSM780NLO confocal laser scanning microscope (Jena, Germany) using 63X objective lens, 405 nm laser to excite **Ir-OC** or **Ir-OA** (emission gain: 520-600 nm), and 647 nm laser to excite MitoTracker™ Deep Red FM (emission gain: 620-696 nm). Live cells were imaged in the CO_2 incubator at 37 °C under humidified atmosphere and 5% CO_2. Airyscan images were obtained to identify the detailed localisation of **Ir-OA**. For co-localisation image with MitoTracker™ Deep Red FM (Mitochondrial membrane), the prepared HeLa cells grown on confocal dish were incubated with culture media of 4 μM **Ir-OA** for 120 min and further incubated with 100 nM MitoTracker for 30 min. Then, the mitochondria of HeLa cells were swelled to identify mitochondrial membrane and matrix by scanning CLSM laser (405 nm, 0.0125 mW). After that, the specific location of **Ir-OA** was images by Airyscan imaging (63X objective lens; 405 nm laser to excite **Ir-OA**, emission gain: 495-550 nm; and 642 nm laser to excite MitoTracker™ Deep Red FM, emission gain: 655-700 nm). For co-localisation image with EGFP in mitochondrial matrix, Mito-EGFP Plasmids staining mitochondrial matrix (see Supplementary Table 3) were transfected into grown cells on confocal dish with TurboFect® Transfection Reagent (Thermo Fisher, R0531). After 24 hours, the HeLa cells were incubated with culture media of 4 μM **Ir-OA** (Culture media:DMSO = 1000:1, v/v) for 120 min and fixed in 4% paraformaldehyde for 15 min at room temperature. Then, the sample was imaged with Airyscan mode of Carl Zeiss LSM980 using 63X objective lens, 405 nm laser to excite **Ir-OA** (emission gain: 550-620 nm), and 488 nm laser to excite EGFPs (500-550 nm). For co-localisation imaging of EGFPs targeting ER, peroxisome, and nucleus with **Ir-OA**, the transfected HEK293T and HeLa cells are prepared as described above. Then, the cells were incubated with culture media of 4 μM **Ir-OA** (Culture media:DMSO = 1000:1, v/v) for 120 min without fixation. The samples were imaged with Carl Zeiss LSM980 using 63X objective lens, 405 nm laser to excite **Ir-OA** (emission gain: 550-650 nm), and 488 nm laser to excite EGFPs (500-550 nm). Then, the Pearson's coefficient r was calculated by image J.

Supplementary Method 6. Intracellular ROS generation assay: H_2DCF-DA assay. 2′,7′-Dichloro-dihydrofluorescein diacetate (H_2DCF-DA) is ROS indicator working inside live cells. The H_2DCF-DA is oxidised to dichlorofluorescein (DCF) emitting strong green fluorescence by reacting with ROS inside cells. For H_2DCF-DA assay, HeLa cells were grown 24 hours before H_2DCF-DA assay and treated with the 4 μM **Ir-OC** and **Ir-OA** for 120 min (Culture media:DMSO = 1000:1, v/v). After washing with DPBS several times, the HeLa cells were further incubated with FBS free DMEM of 20 μM H_2DCF-DA for 60 min and were washed again. Then, the HeLa cells were irradiated by LED array (λ = 400 nm, 170 mJ cm^{-2}), and imaged in live cell incubator with Carl Zeiss LSM780NLO confocal laser scanning microscope (Jena, Germany) using 20X objective lens and 488 nm laser to excite DCF (emission gain: 500-550 nm). This experiment was repeated for negative controls conditions (Light irradiation without iridium complex).

Supplementary Method 7. Cell viability test (live or dead assay, MTT assay, and CCK-8 assay). The live or dead assay was conducted to confirm the photoinduced cell-killing effect of **Ir-OC** and **Ir-OA**. For the live or dead assay, HeLa cells were grown on the confocal dish for 24 hours. The HeLa cells were incubated with culture media of 8 μM **Ir-OC** and **Ir-OA** for 120 min (Culture media:DMSO = 1000:1, v/v), and culture media was changed. Then, the HeLa cells were irradiated by LED array (λ = 400 nm, 255 mJ cm^{-2}). After further incubation for 24 hours, the culture media were changed to new one with 1.5 μM of propidium iodide (Thermo Fisher, P1304MP) for staining dead cells and 2.5 μM of calcein AM (Thermo Fisher, C1430) for stain live cells. The samples were imaged with Carl Zeiss LSM780NLO confocal laser scanning microscope (Jena, Germany) using 20X objective lens, 488 nm laser to excite calcein AM (emission gain: 500-550 nm), and 647 nm laser to excite propidium iodide (emission gain: 620-680 nm). Additionally, 6 groups of HeLa cells were grown on the 6-well plate for quantitative analysis of live or dead assay using flow cytometry (Ir-, **Ir-OA** and **Ir-OC** of dark or light-

irradiated condition). Cells were incubated with the culture media containing 8 μM **Ir-OC** or **Ir-OA** for 120 min. At the same time, the control groups without treating the iridium complexes were incubated. After changing into fresh media, three groups of cells (Ir- hv+, **Ir-OA** hv+ and **Ir-OC** hv+) were irradiated by LED array (λ = 400 nm, 255 mJ cm^{-2}) and further incubated for 24 hours. The adherent cells were harvested and washed with cold PBS, followed by suspending in PBS containing 1.5 μM of propidium iodide and 2.5 μM of calcein AM. After 15 min incubation at 37 °C, the stained cells were immediately analysed by flow cytometry, measuring the fluorescence emission at 527 nm (excitation 488 nm) for calcein AM and 660 nm for PI. We allocated all cells into the experimental groups because we had to count dead cells which could not maintain their internal complexity. Even though little amount of cell debris can be included to the experiments, we confirmed significant different results between control groups and experimental group. The whole procedure was conducted in the darkroom and the cells were kept in ice during flow cytometry. The cells for apoptosis assay were treated in the same way with that prepared for flow cytometry of live or dead assay and harvested as described in the protocols of *Invitrogen*. After 6-hour additional incubation after light irradiation, the adherent cells were harvested and washed with cold PBS. Then, the cells were centrifuged and resuspended in 100 ul of 1X annexin V binding buffer with 5 μL Alexa Fluor® 488-annexin V and 1 μL 100 μg/mL PI working solution per each condition. After 15 min incubation in room temperature, 400 μL of annexin V binding buffer was additionally added and the cell were analyzed using flow cytometry measuring fluorescence emission at 527 nm (excitation 488 nm) for Alexa Fluor® 488-annexin V and 660 nm for PI. The starting cells were selected by the preliminary FSC/SSC gates to find reasonable size cells with internal complexity. We choose cells with positive FSC and positive SSC, and discarded cells with extremely high SSC or FSC. The boundaries between positive/negative were 7 X 10^4, 7 X 10^4 (Annexin V/PI) as described below. The FACS data was processed by the BD FACSuite software (ver. 1.0.6). To quantify the dark-toxicity and photo-toxicity of **Ir-OC** and **Ir-OA**, the MTT assay was conducted. HeLa cells were grown in Dulbecco's Modified Eagle Medium containing 10% fetal bovine serum (FBS) and 1% penicillin-streptomycin (10,000 u/mL). HeLa cells were cultured in a humidified incubator at 37 °C with 5% CO_2. Photodynamic effect of **Ir-OA** and **Ir-OC** was quantified by MTT assay [MTT = 3-(4,5-dimethyl-2-thiazolyl)-2,5-diphenyl-2H-tetrazolium bromide]. 1.5 x 10^5 cells/mL of Cells were seeded in a 96-well plate and incubated overnight. Then, 1 μL of **Ir-OA** and **Ir-OC** in different concentrations were added to each well containing the 100 μL of cell culture media, so the final concentrations of iridium complexes were set to be the power of 2 from 0.5 μM to 64 μM. After 2 hours incubation, the media containing iridium complexes was replaced by 100 μL of fresh media and predetermined intensities of 400 nm blue light (0, 0.085, 0.170, 0.255 J cm^{-2}) was given to cell plate. For followed 24 hours, the cells were incubated at 37 °C with 5% CO_2 in the dark. MTT (25 μL with 5 mg/mL PBS, pH 7.4) was added to each well and cells were further incubated for 3 hours. The old medium containing unreacted MTT was removed carefully and formazan produced by living cells was dissolved in 100 μL of solubilisation buffer [DMF (50% v/v), sodium dodecyl sulfate (10% w/v), 99.9% AcOH (0.4% v/v), and 1 N HCl (0.4% v/v)] overnight at room temperature. The absorbance of formazan at 570 nm was measured using a SpectraMax M5 microplate reader (Molecular Devices, Sunnyvale, CA, USA). Cell viability was then determined relative to the control condition (average value of the absorbance of cells containing 1% of DMSO). The iridium complex treating procedure of cell counting kit CCK-8 assay is same with MTT assay. The difference is, after 24-hour incubation of light irradiated HeLa cells, the 10 μL of CCK-8 solution was treated in each well. After 4-hour incubation at 37 °C, the absorbance of formazans were measured at 460 nm using a SpectraMax M5 microplate reader.

Supplementary Method 8. Time-correlated single photon counting (TCSPC). The lifetime of **Ir-OA** was recorded with a time-correlated single photon counting (TCSPC) setup (FluoTime300, PicoQuant). For different viscosity, each sample was prepared as 20 μM concentration in different methanol/glycerol ratio (5, 20, 40, 60, 80, and 100, v/v%). For lifetime test by bovine serum albumin (BSA) concentration, BSA was dissolved in PBS:DMSO=99:1 solvent with the same concentration of the photosensitiser. The lifetime was measured titrating BSA concentration from 4.00 mg/ml to 0.0156 mg/ml for every quarter value. The samples were photoexcited using a 450 nm cw and pulsed diode laser head (LDH-D-C-450) coupled with a laser diode driver (PDL 820, PicoQuant) with a <70 ps pulse width and 196 kHz–40 MHz repetition rate. A TCSPC module (PicoHarp 300E, PicoQuant) with a photomultiplier tube (PMA-C 182-N-M, PicoQuant) was used for the time-resolved PL signal detection. The spectra were deconvoluted with fitting software (FluoFit, PicoQuant) to calculate corresponding PL lifetimes.

Supplementary Method 9. Mitochondrial viscosity monitoring. The mitochondrial viscosity change was monitored by phosphorescence lifetime imaging microscopy (PLIM). HeLa cells were cultured on two confocal dishes for 24 hours and were incubated with **Ir-OA** (Culture media:DMSO = 1000:1, v/v) for 2 hours respectively. One dish was irradiated by LED array (λ = 400 nm, 170 mJ cm^{-2}), and the other one was blocked from light. Time-resolved fluorescence study was carried out using a confocal microscope (MicroTime-200, Picoquant, Germany) with a 40× objective. The lifetime measurements were performed at the Korea Basic Science Institute (KBSI),

Daegu Center, Korea. A single-mode pulsed diode laser (375 nm with 30 ps pulse width and 0.2~0.6 μW average power) was used as an excitation source. A dichroic mirror (Z375RDC, AHF), a longpass filter (HQ405lp, AHF), a 75 μm pinhole, a longpass filter (FEL0550, Thorlabs) and an avalanche photodiode detector (PDM series, MPD) was used to collect emission photons from the samples. Fluorescence lifetime images consisted of 200×200 pixels were recorded using the time-tagged time-resolved (TTTR) data acquisition method. The acquisition time of each pixel was 1 ms. Exponential fitting for the obtained fluorescence decays was accomplished using the Symphotime-64 software (Ver. 2.2).

Supplementary Method 10. Mitochondrial depolarisation monitoring. Mitochondrial depolarisation was monitored by CLSM Airyscan 2 imaging. HeLa cells were grown on confocal dishes for 24 h, and incubated with culture media of 4 μM **Ir-OA** for 120 min (Culture media:DMSO = 1000:1, v/v). After washing process, the cells were irradiated by LED array (λ = 400 nm, 170 mJ cm^{-2}) and were further incubated for 0, 10, 45, 90, and 180 min to investigate mitochondrial depolarisation time. Then, the cells were imaged with Carl Zeiss LSM980 using 63X objective lens, 405 nm laser to excite **Ir-OA** (emission gain: 573-620 nm, phosphorescence, 420-480 nm, fluorescence). To monitor mitochondrial depolarisation in real time, HeLa cells were prepared as described above. The cells were irradiated by the 405 nm laser of LSM980 for 60 seconds and were imaged during irradiation (Laser power: 1.0%, 14 mW). All images were processed with Carl Zeiss ZEN3.0 and MetaMorph software (ver. 7) for transformation to ratiometric images. All live cells were imaged in the CO_2 incubator at 37 °C under humidified atmosphere and 5% CO_2.

Supplementary Method 11. Western blot for protein photo-crosslinking. HEK293T or HeLa cells were incubated with DMEM (containing 10% FBS, 50 units/mL penicillin, and 50 μg/mL streptomycin) under 37 °C and 5% CO_2 condition. HEK293T was transfected with Turbofect® transfection reagent (Thermo Fisher Scientific, MA, USA) and four different EGFP constructs for each cell organelles (Mito-EGFP; mitochondria, PTBP1-EGFP; nucleus, Sec61b-EGFP; endoplasmic reticulum, PEX16-EGFP; peroxisome) in 70% confluency. 5 μM **Ir-OA** was incubated (Culture media:DMSO = 1000:1, v/v) for 1 hour after 18 hours. Ir(III) complex incubated cells were irradiated for 5 minutes with LED array (400 nm, 1.28 J cm^{-2}) after two times washing with Dulbecco's phosphate-buffered saline (DPBS) (GibcoTM, CA, USA). The lysis was done with RIPA protein extraction solution (50 mM Tris-HCl pH 7.5, 150 mM NaCl, 1% NP-40, 0.5% deoxycholic acid, 0.1% SDS, 1 mM PMSF) containing Halt™ protease inhibitor cocktail (Thermo Fisher Scientific, MA, USA). The lysis solution of whole proteins was obtained from supernatant by centrifuge (16,000 xg, 10 minutes, 4°C). Protein denaturation was proceeded for 5 minutes with 90°C in heat block and subsequent 4°C cooling step was followed for 5 minutes after mixing with SDS-PAGE loading buffer (Biosesang, Gyeonggi-do, Korea). Loading quantity was accurately quantified by bicinchoninic acid (BCA) assay (Thermo Fisher Scientific, MA, USA). That lysis solution was separated by SDS-PAGE gel electrophoresis and separated proteins on the gel were transferred to the nitrocellulose membrane (Pall corporation, NY, USA). Successful transfer of the proteins was identified by Ponceau S (0.1% (w/v) Ponceau S in 5% acetic acid/water) staining and staining was removed with 0.1% Tween-20 in Tris-buffered saline (TBST). Proteins on the membrane was firstly blocked with the 2% skim milk containing TBST solution for 1 hour. The membrane was incubated for 30 minutes with 3000:1 diluted GFP tag monoclonal antibody (GF28R) (Thermo Fisher Scientific, MA, USA) as primary antibody. Not attached non-specific antibody was strictly washed 3 times with TBST for 5 minutes each. Anti-mouse HRP as secondary antibody was incubated for 30 minutes and 3 times washing was followed ut supra. Western blot chemiluminescence signal was recorded by G:BOX Chemi XRQ (Syngene, Cambridge, UK) after development with Clarity reagent (Bio-rad, CA, USA).

Supplementary Method 12. Line-cut analysis of western blot for cross-linking and calculation of correlation value. Line-cut analysis was proceeded with ImageJ software (National Institute of Health, NIH) and detailed procedure referred to previously reported paper.[4] The background of western blot for photo-crosslinking in Figure 4e was properly subtracted in the software (ball-point size: 20 pt). For western blot signal quantification, same length of the line was drawn on each lane and profiled the line-cut intensity along the line. In addition, similarity between two different line cut profiling with or without photo-irradiation was calculated as correlation index (C_{ij}) according to the equation presented below. Photo-crosslinking efficiency (η_c) was obtained by second equation including C_{ij}, from which discrepancy between two line-cut profiling results with or without photo-irradiation was quantified.

$$C_{ij} = \frac{\frac{1}{N}\sum_{k=1}^{N} I_i(k) I_j(k)}{\sqrt{\frac{1}{N}\sum_{k=1}^{N} I_i(k)^2}\sqrt{\frac{1}{N}\sum_{k=1}^{N} I_j(k)^2}} \quad (1)$$

$$\eta_c(\%) = \left(1 - C_{ij}\right) \times 100\,\% \quad (2)$$

Supplementary Method 13. Mitochondrial membrane potential assay (TMRE assay).
To confirm the mitochondrial depolarisation by photoactivation of **Ir-OA**, Tetramethylrhodamine ethyl ester (TMRE), a conventionally used mitochondrial membrane potential (MMP) indicator, was used. All HeLa cells were cultured on confocal dishes for 24 h and incubated with culture media of 4 μM **Ir-OA** (Culture media:DMSO = 1000:1, v/v) for 120 min. After several washing process with DPBS, the cells were further incubated with 200 nM TMRE for 30 min. Then, some dishes were irradiated by LED array (λ = 400 nm, 170 mJ cm^{-2}), the others were blocked from light. The samples were imaged with Carl Zeiss LSM780NLO confocal laser scanning microscope (Jena, Germany) using 63X objective lens, 405 nm laser to excite **Ir-OA** (emission gain: 520-560 nm), and 562 nm laser to excite TMRE (emission gain: 600-696 nm). The intensity of the image was integrated with Carl Zeiss ZEN2012 software (UNIST Olympus Biomed Imaging Center, Ulsan, Republic of Korea). All live cells were imaged in the CO_2 incubator at 37 °C under humidified atmosphere and 5% CO_2.

Supplementary Method 14. Mitochondrial morphology monitoring. To monitor morphological change of mitochondria in response to oxidative stress, the time-series Airyscan 2 images were obtained. Mito-EGFP Plasmids were transfected into grown cells on a confocal dish to stain mitochondrial matrix. After 24 hours, the HeLa cells were incubated with culture media of 4 μM **Ir-OA** (Culture media:DMSO = 1000:1, v/v) for 120 min. The cells were washed with DPBS several times and were imaged for 340 seconds with time-series Airyscan 2 mode of Carl Zeiss LSM980 using 63X objective lens, 405 nm laser to give oxidative stress by photoactivation of **Ir-OA** (power: 1.0%, 14 mW), and 488 nm laser to excite EGFPs (emission gain: 500-550 nm) All live cells were imaged in the CO_2 incubator at 37 °C under humidified atmosphere and 5% CO_2.

Supplementary Figures

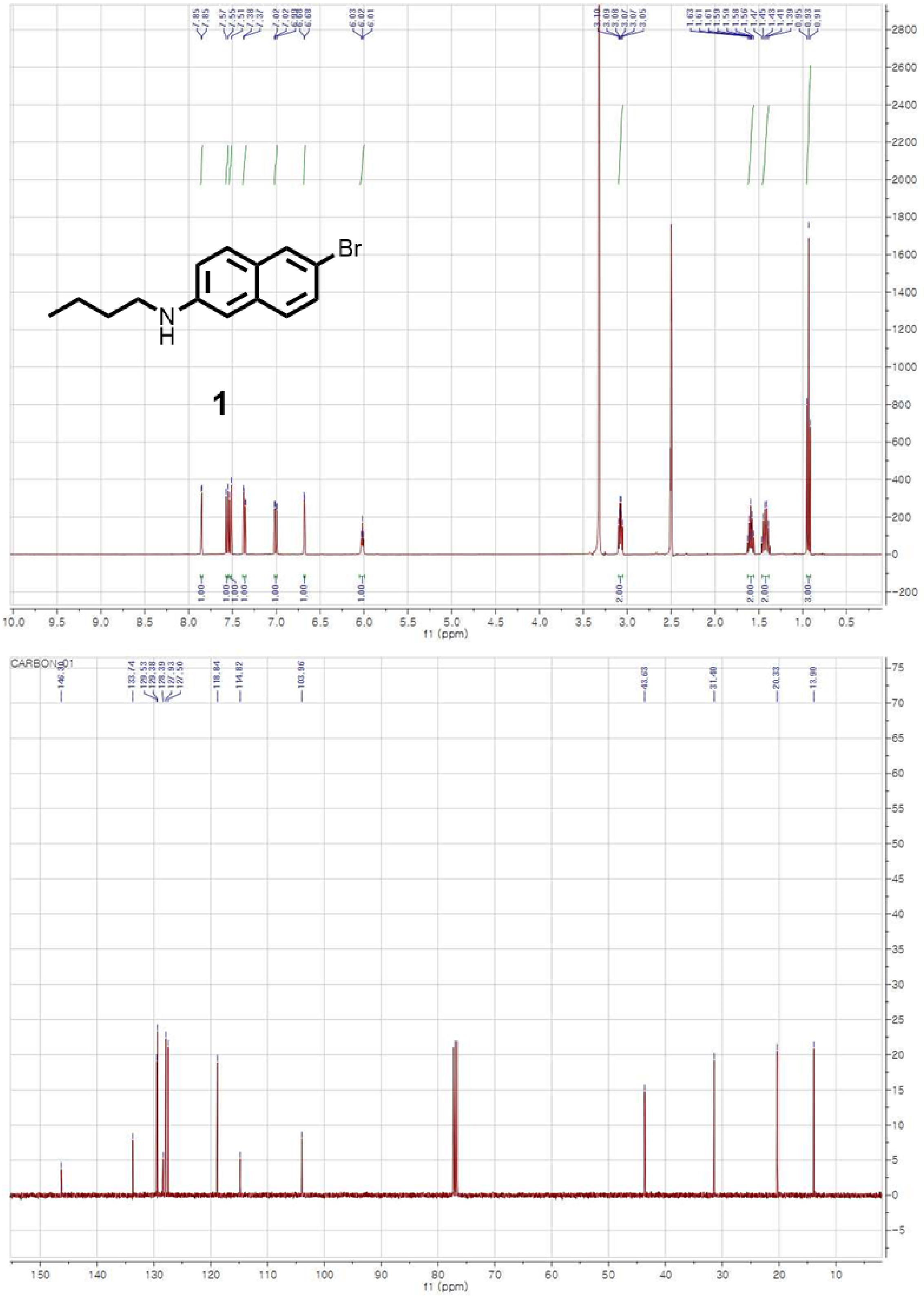

Supplementary Figure 1. ^{1}H NMR (400 MHz, d_6-DMSO) and ^{13}C NMR (100 MHz, chloroform-d) spectrum of Compound 1.

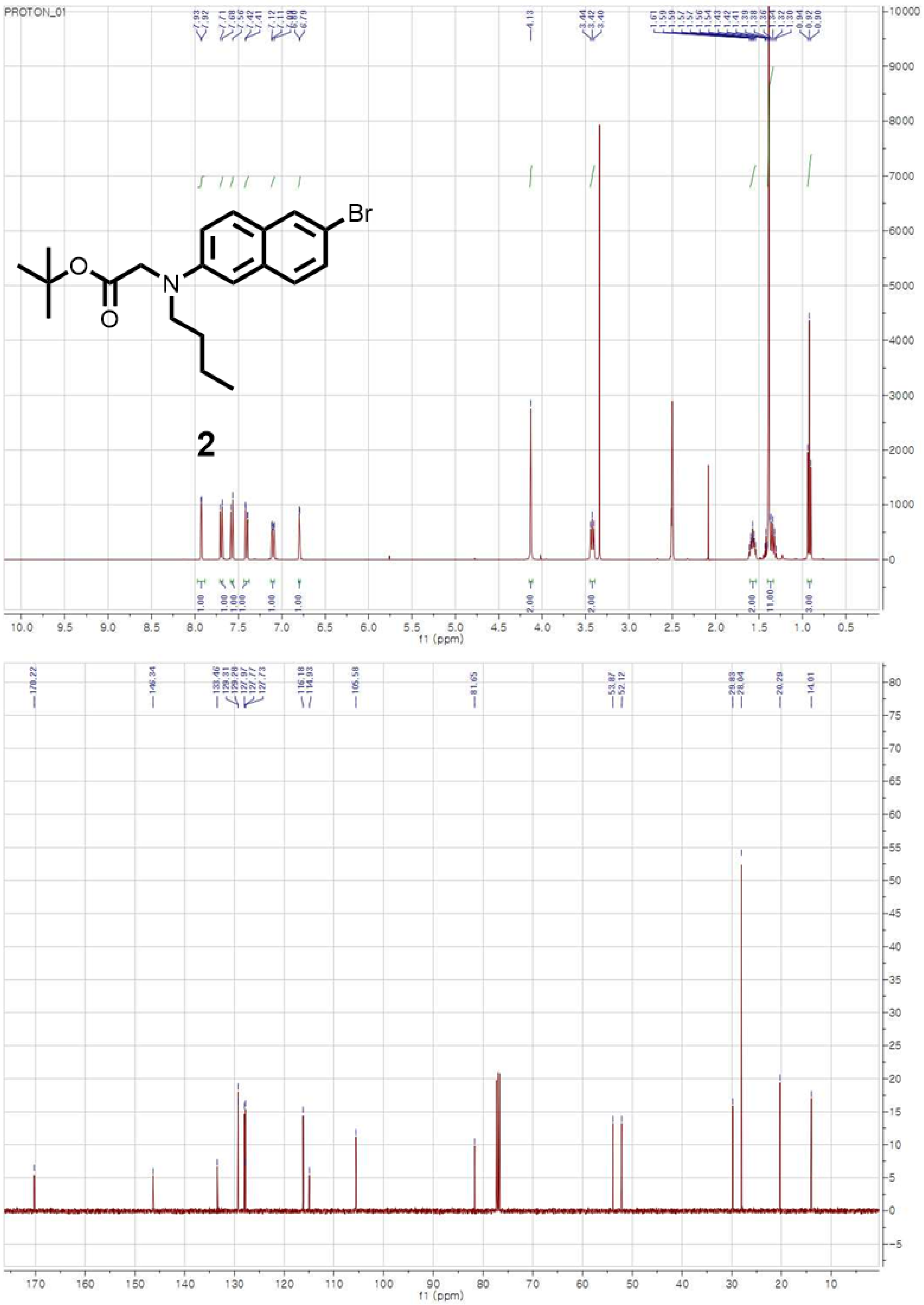

Supplementary Figure 2. ^{1}H NMR (400 MHz, d_6-DMSO) and ^{13}C NMR (100 MHz, chloroform-d) spectrum of Compound 2.

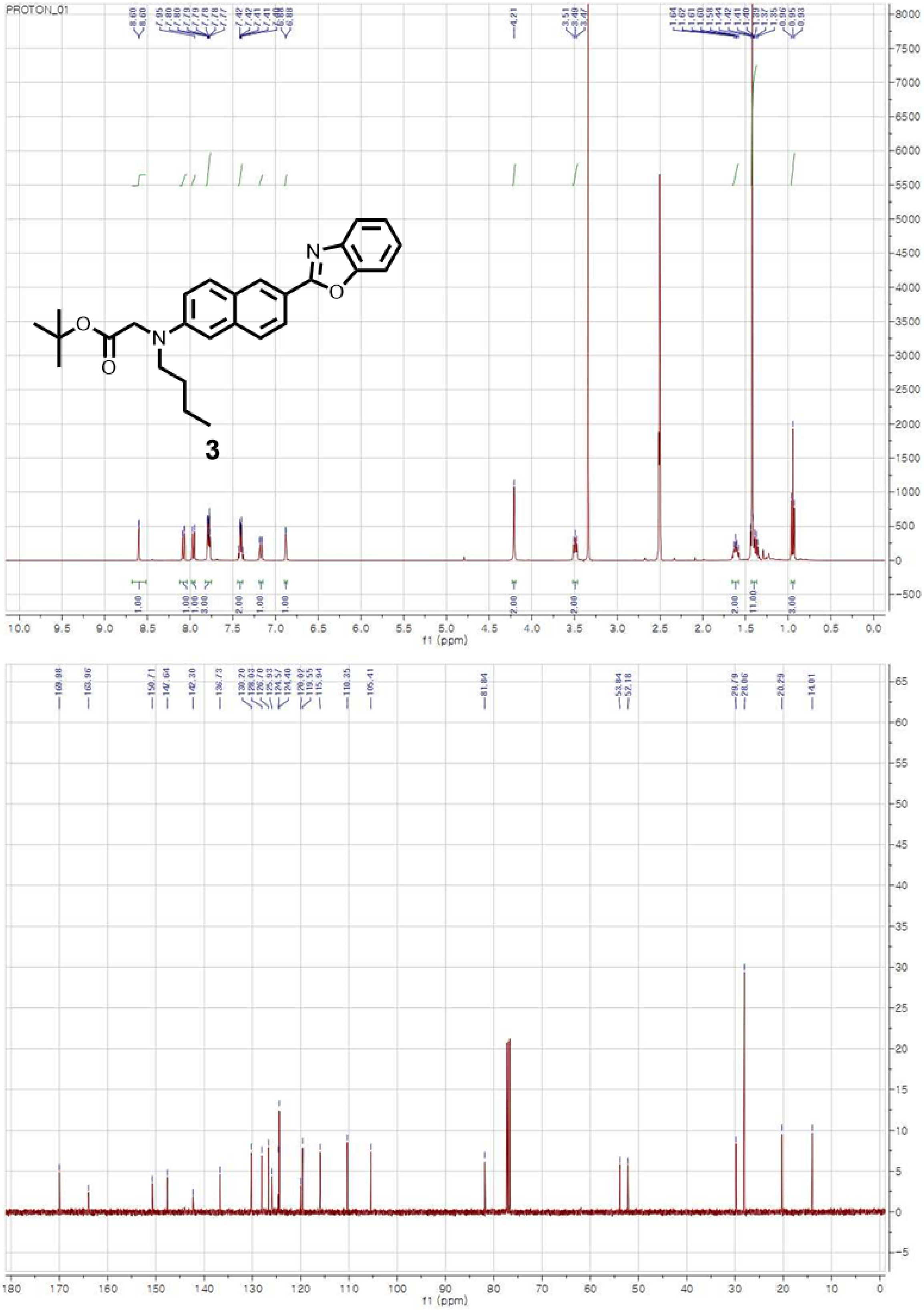

Supplementary Figure 3. ^{1}H NMR (400 MHz, d_6-DMSO) and ^{13}C NMR (100 MHz, chloroform-d) spectrum of Compound 3.

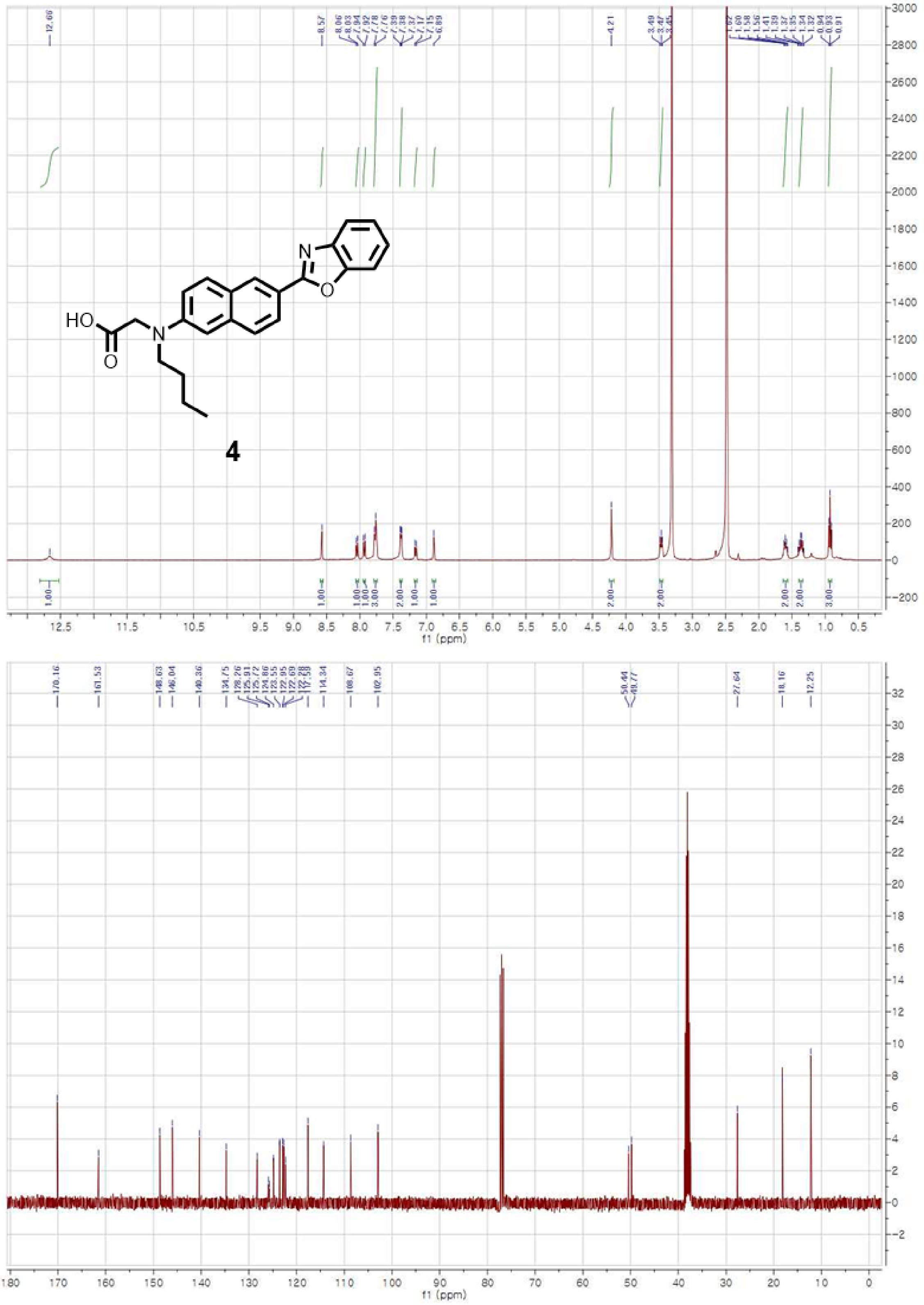

Supplementary Figure 4. ^{1}H NMR (400 MHz, d_6-DMSO) and ^{13}C NMR (100 MHz, chloroform-d) spectrum of Compound 4.

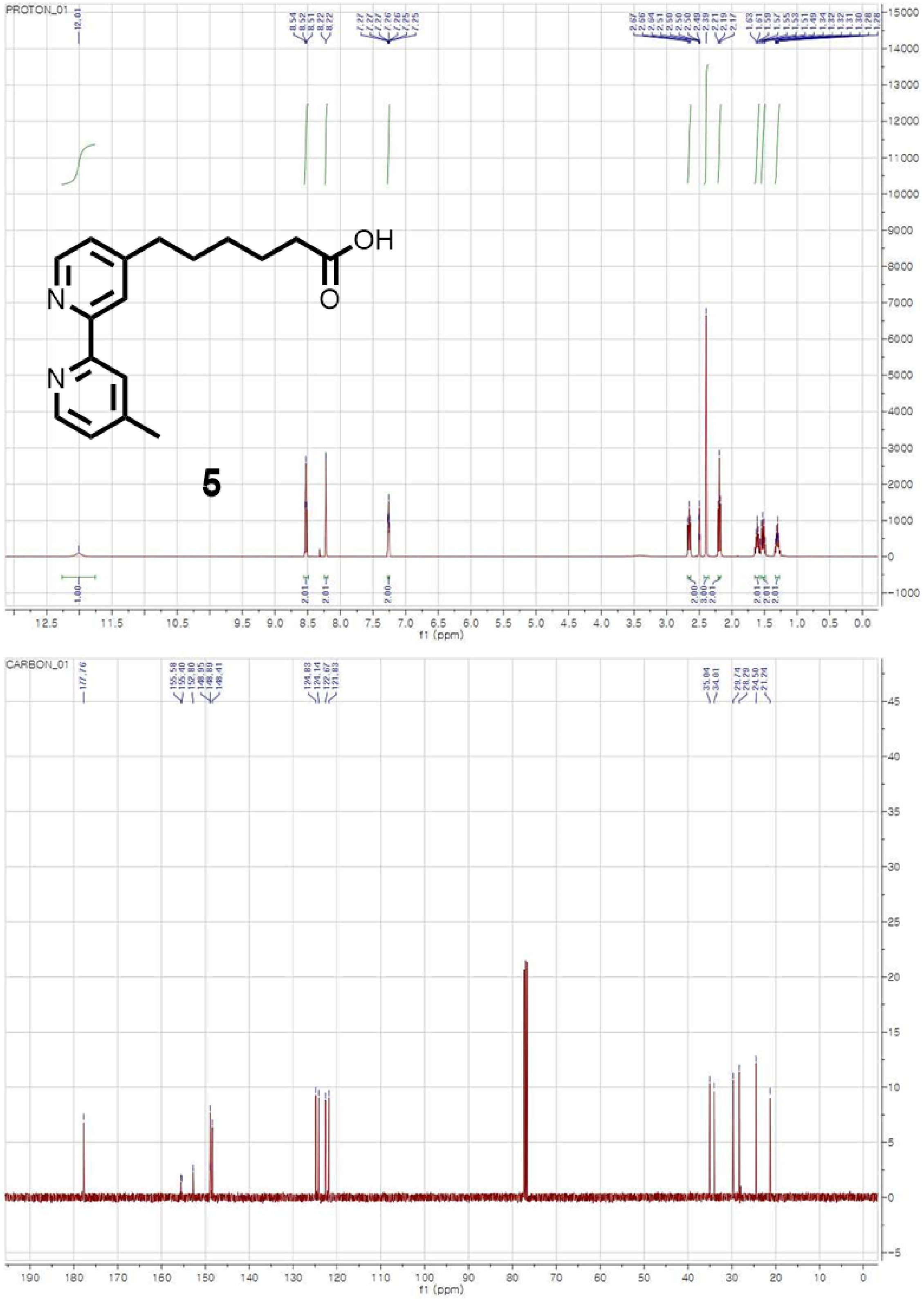

Supplementary Figure 5. ^{1}H NMR (400 MHz, d_6-DMSO) and ^{13}C NMR (100 MHz, chloroform-d) spectrum of Compound 5.

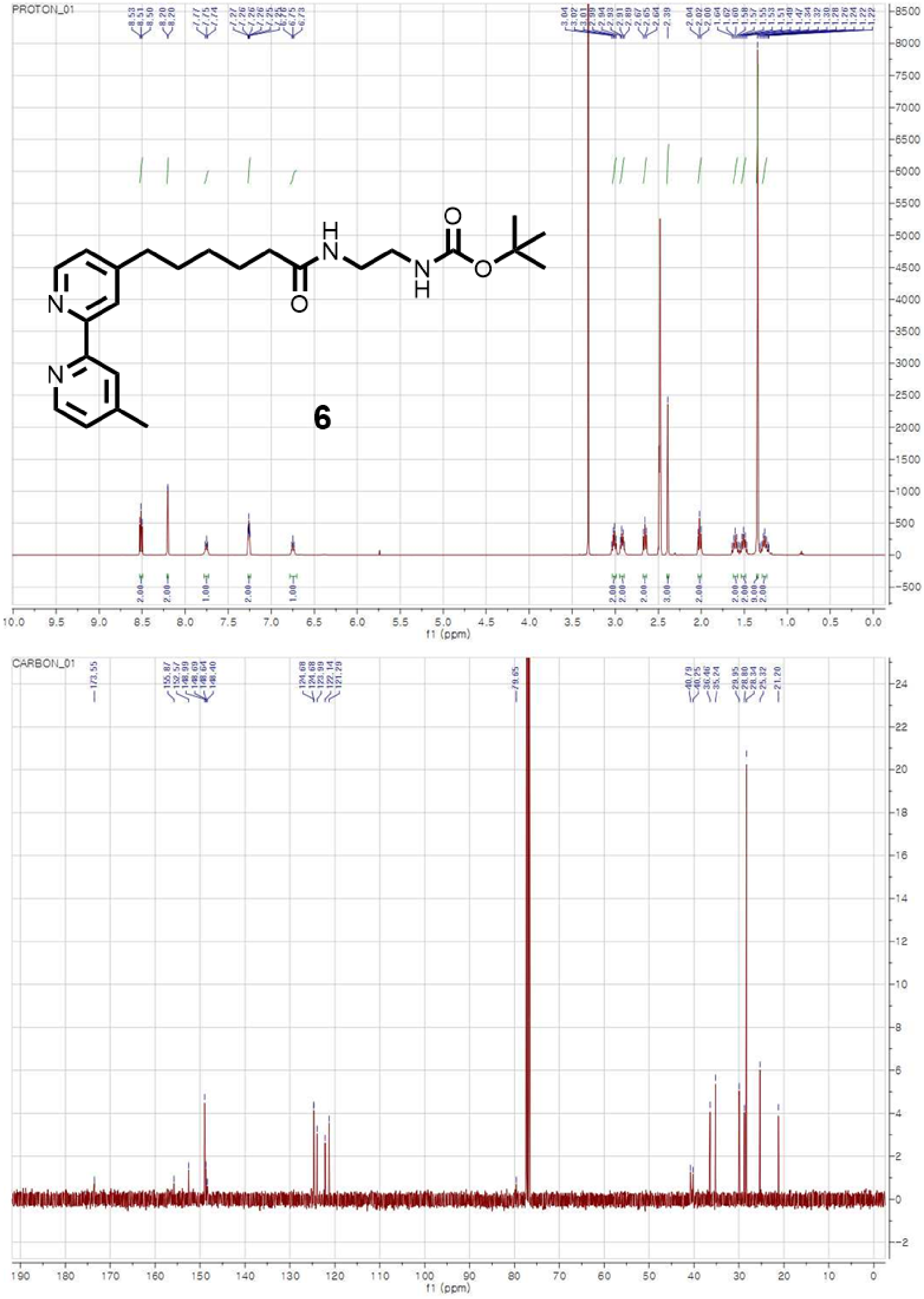

Supplementary Figure 6. ^{1}H NMR (400 MHz, d_6-DMSO) and ^{13}C NMR (100 MHz, chloroform-d) spectrum of Compound 6.

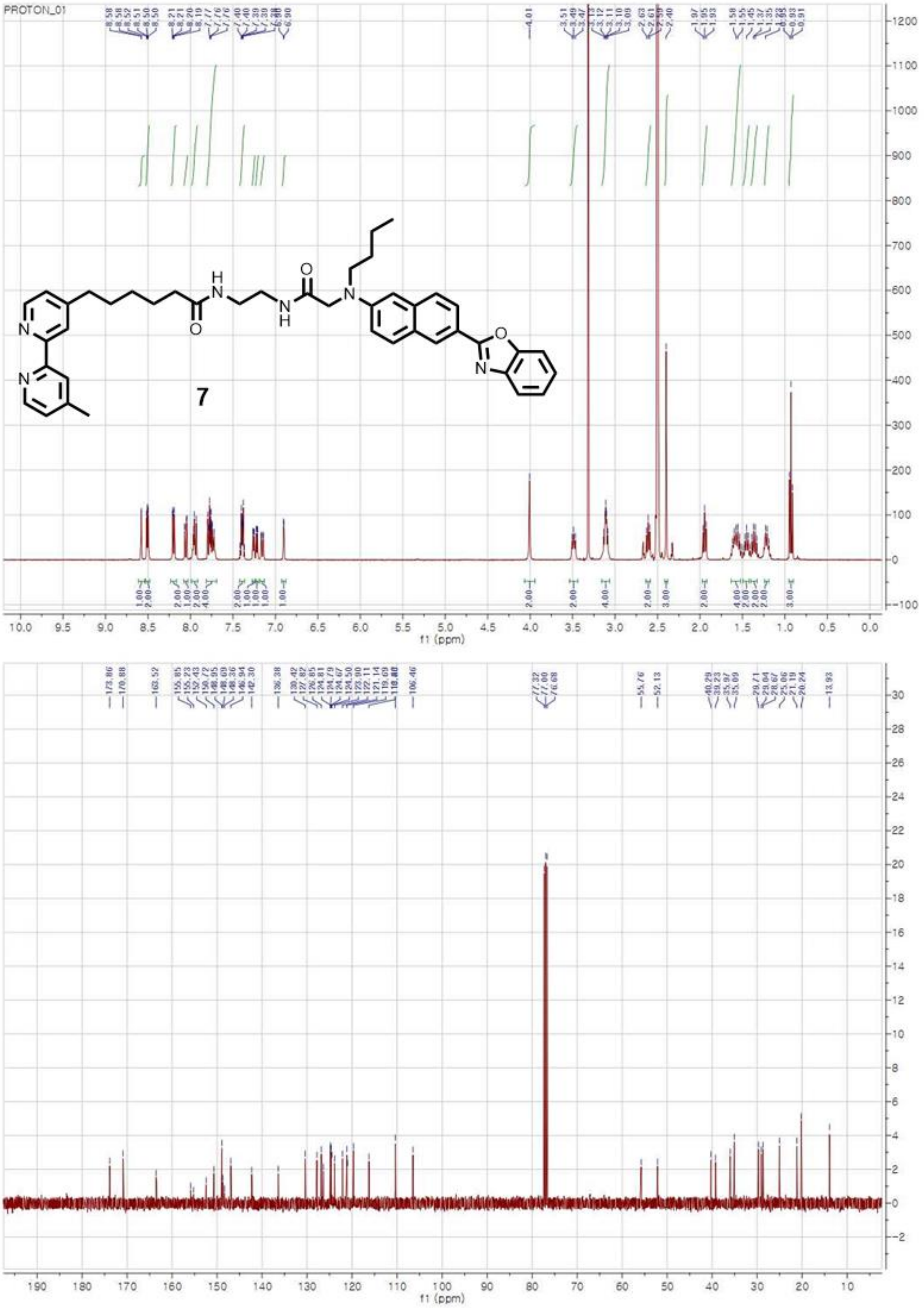

Supplementary Figure 7. ^{1}H NMR (400 MHz, d_6-DMSO) and ^{13}C NMR (100 MHz, chloroform-d) spectrum of Compound 7.

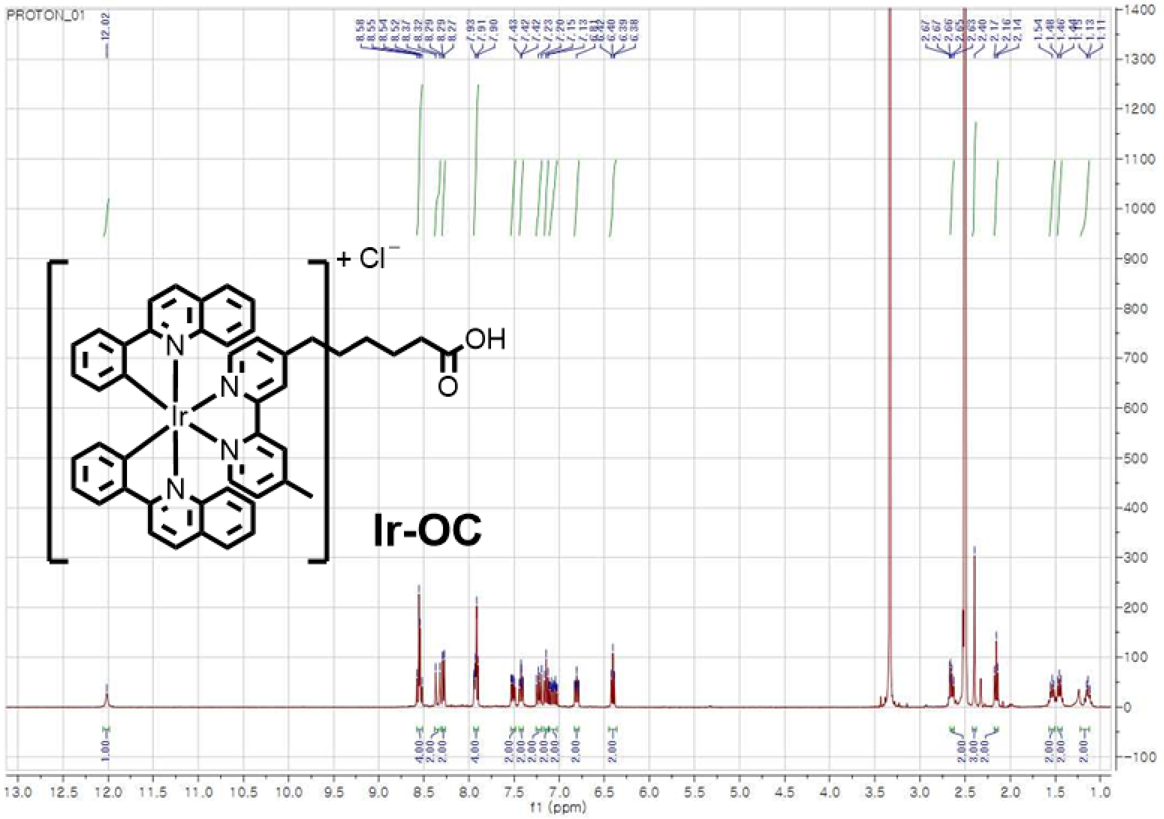

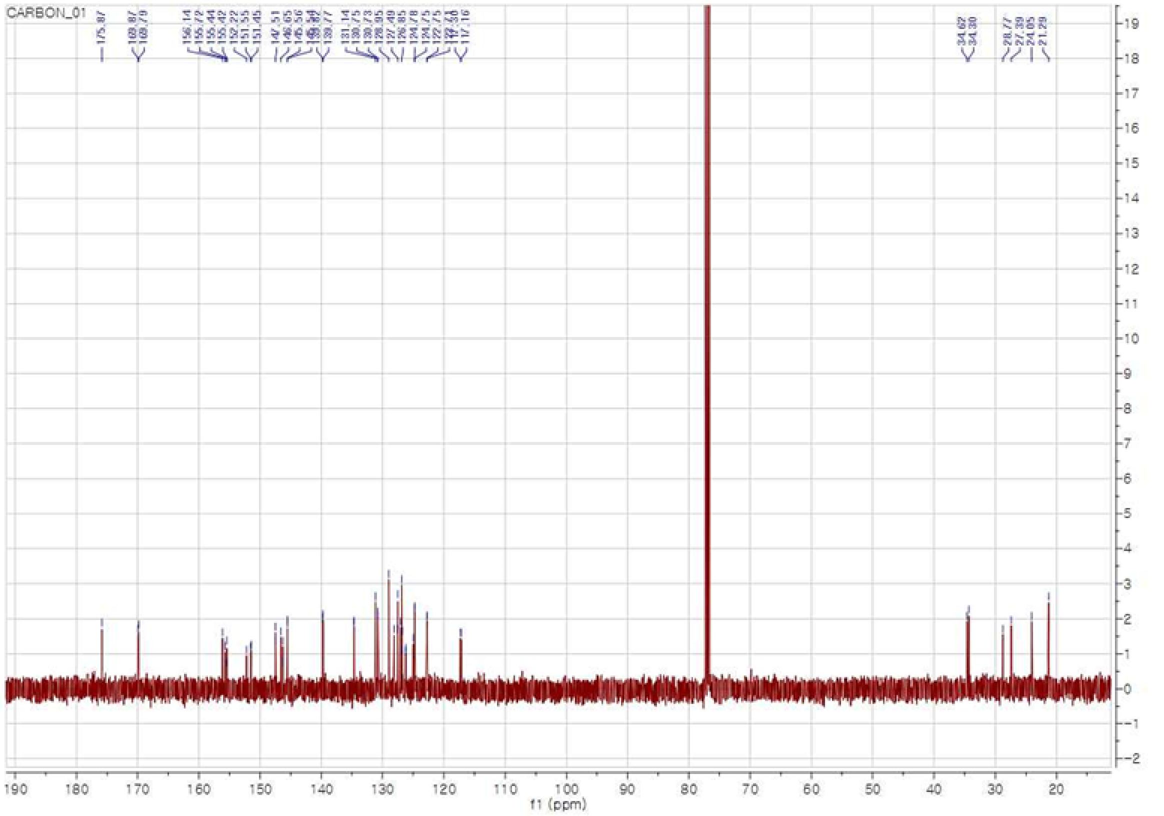

Supplementary Figure 8. ^{1}H NMR (400 MHz, d_6-DMSO) and ^{13}C NMR (100 MHz, chloroform-d) spectrum of **Ir-OC**.

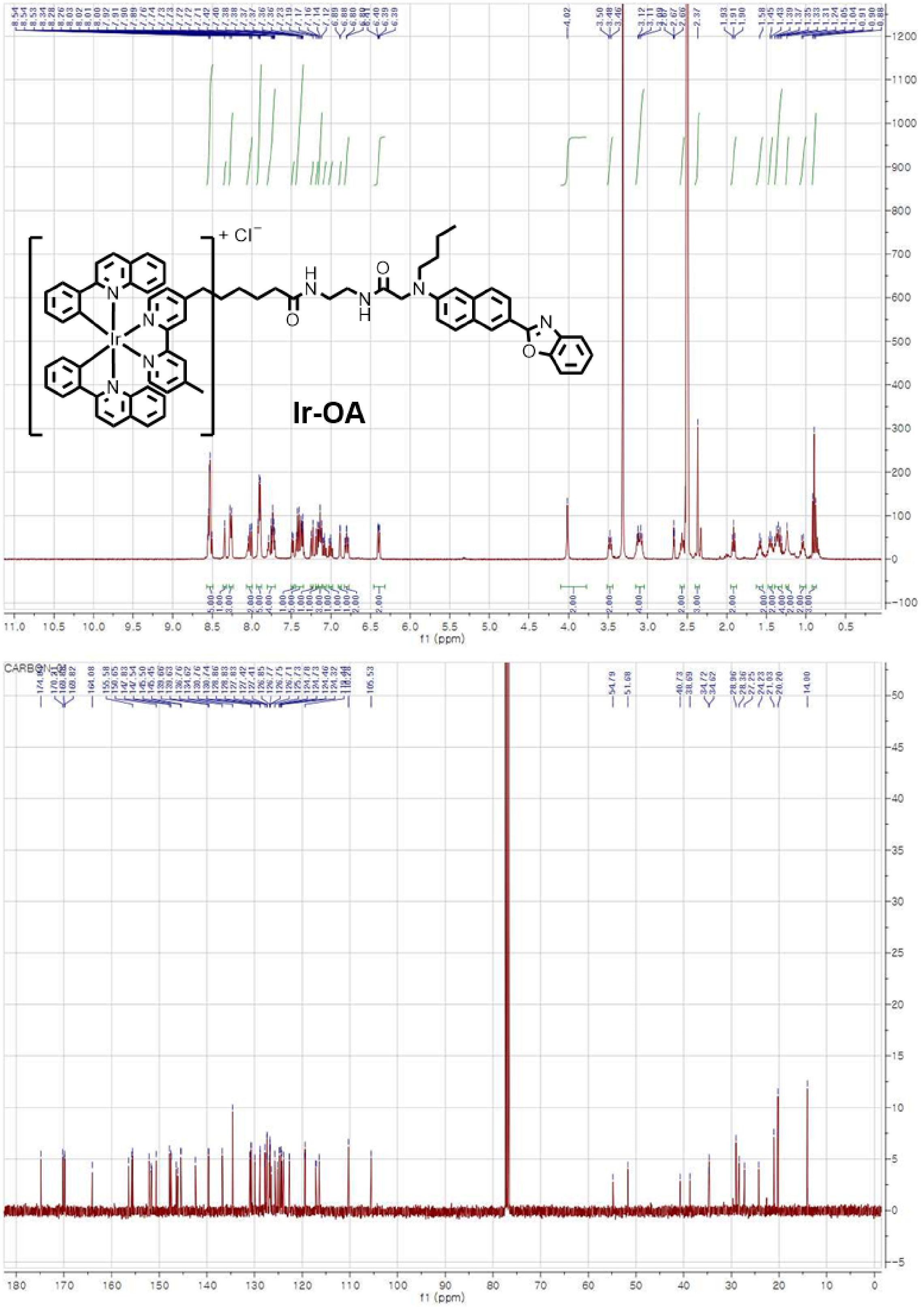

Supplementary Figure 9. ^{1}H NMR (400 MHz, d_6-DMSO) and ^{13}C NMR (100 MHz, chloroform-d) spectrum of **Ir-OA**.

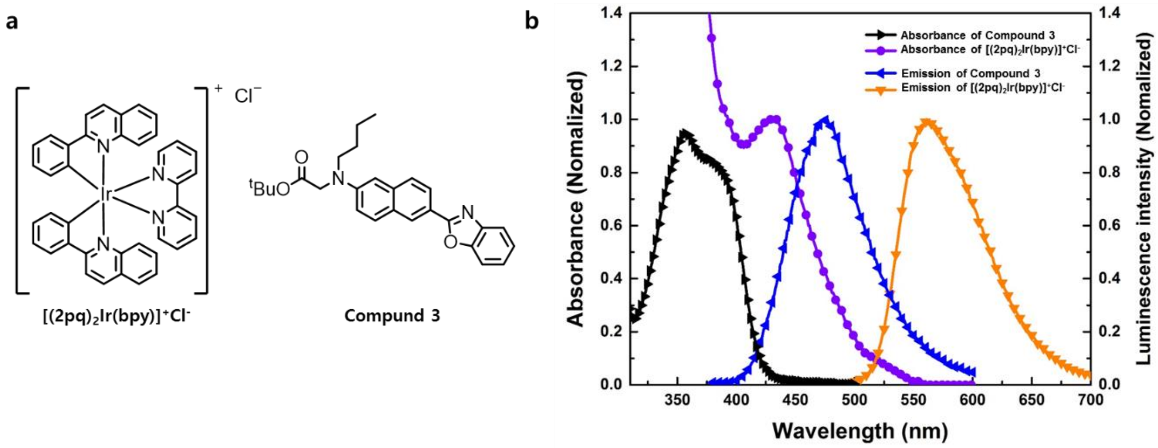

Supplementary Figure 10. Absorption and emission spectra of energy donor and acceptor. **a.** the molecular structure of the $[(2pq)_2Ir(bpy)]^+Cl^-$ (energy acceptor) and the compound 3 (energy donor). **b.** UV-vis absorption spectra of $[(2pq)_2Ir(bpy)]^+Cl^-$ (purple) and compound 3 (black); emission spectra (λ_{ex} = 400 nm) of $[(2pq)_2Ir(bpy)]^+Cl^-$ (orange) and compound 3 (blue). Note that the absorption spectrum of $[(2pq)_2Ir(bpy)]^+Cl^-$ and emission spectrum of compound 3 overlaps well. Conditions: [Donor or acceptor] = 20 μM in H_2O:DMSO = 99:1 (v/v%).

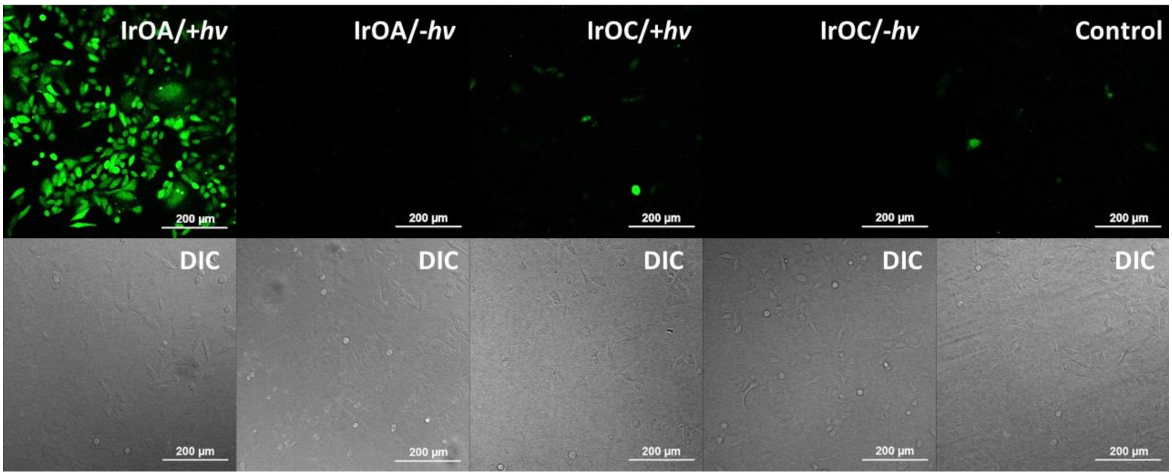

Supplementary Figure 11. H_2DCF-DA assay for identification of ROS generation inside live cells. The green fluorescence of DCF turned on after photoactivation of **Ir-OA** or **Ir-OC**. Conditions: [**Ir-OA** or **Ir-OC**] = 4 μM, [H_2DCF-DA] = 20 μM, 400 nm light LED array (0.170 J cm^{-2}), control: 400 nm light irradiated HeLa cells without incubation of iridium complexes. The experiment was repeated three times independently, and each experiment showed similar results.

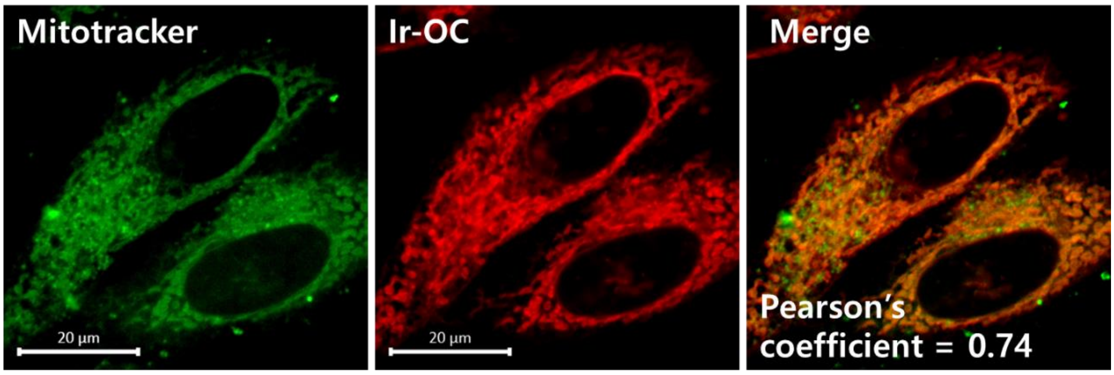

Supplementary Figure 12. Localisation of **Ir-OC** in the living cells. Confocal images of **Ir-OC** with MitoTracker. Phosphorescence of **Ir-OC** (green), fluorescence of MitoTracker (Red) and merge image (λ_{ex} = 405 nm for **Ir-OC**, λ_{ex} = 647 nm for MitoTracker® Deep Red FM). Pearson's coefficient was calculated by Image J software. Conditions: [**Ir-OC**] = 4 µM, [MitoTracker Deep Red] = 100 nM. The experiment was repeated three times independently, and each experiment showed similar results.

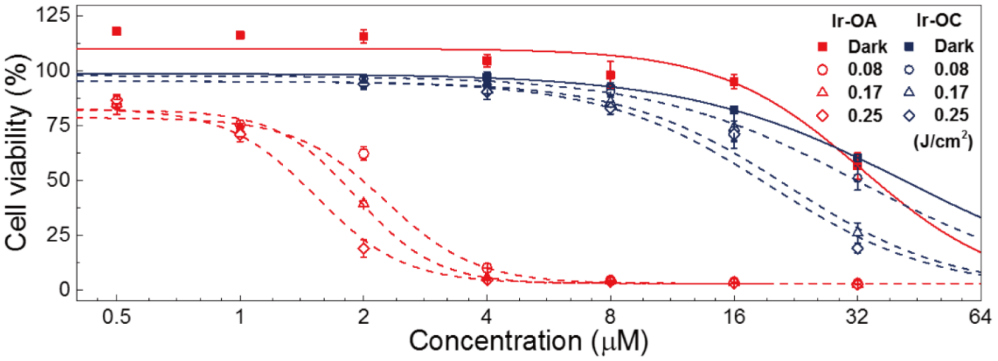

Supplementary Figure 13. MTT-assay for HeLa cells with **Ir-OA** and **Ir-OC**. Quantitative analysis of cytotoxicity of **Ir-OA** and **Ir-OC** with or without light irradiation. The assays were triplicate. Conditions: Ir(III) complexes incubation time = 2 hours, light source = 400 nm light LED array, light dose: 0.08, 0.17, and 0.25 J cm^{-2}. Data are represented as mean ± s. d. (n=4). Source data are provided as a Source Data file.

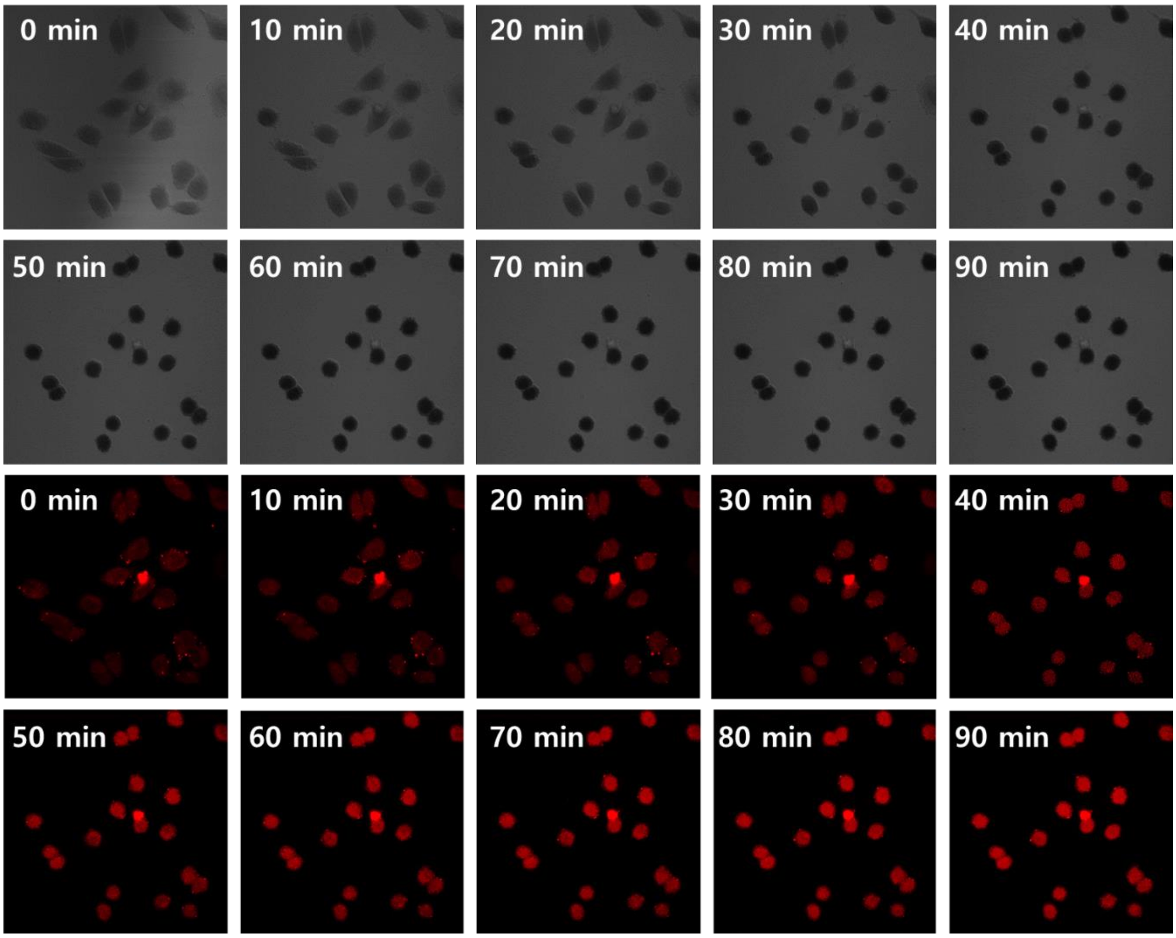

Supplementary Figure 14. Real time imaging for in vitro photodynamic therapy. After light irradiation, the cellular morphology and the red signal of propidium iodide (PI) were monitored for 90 minutes. The PI stains dead cells. Conditions: [PI] = 1.5 μM, light source = 400 nm light LED array, light dose = 0.170 J cm^{-2}. The experiment was repeated three times independently, and each experiment showed similar results.

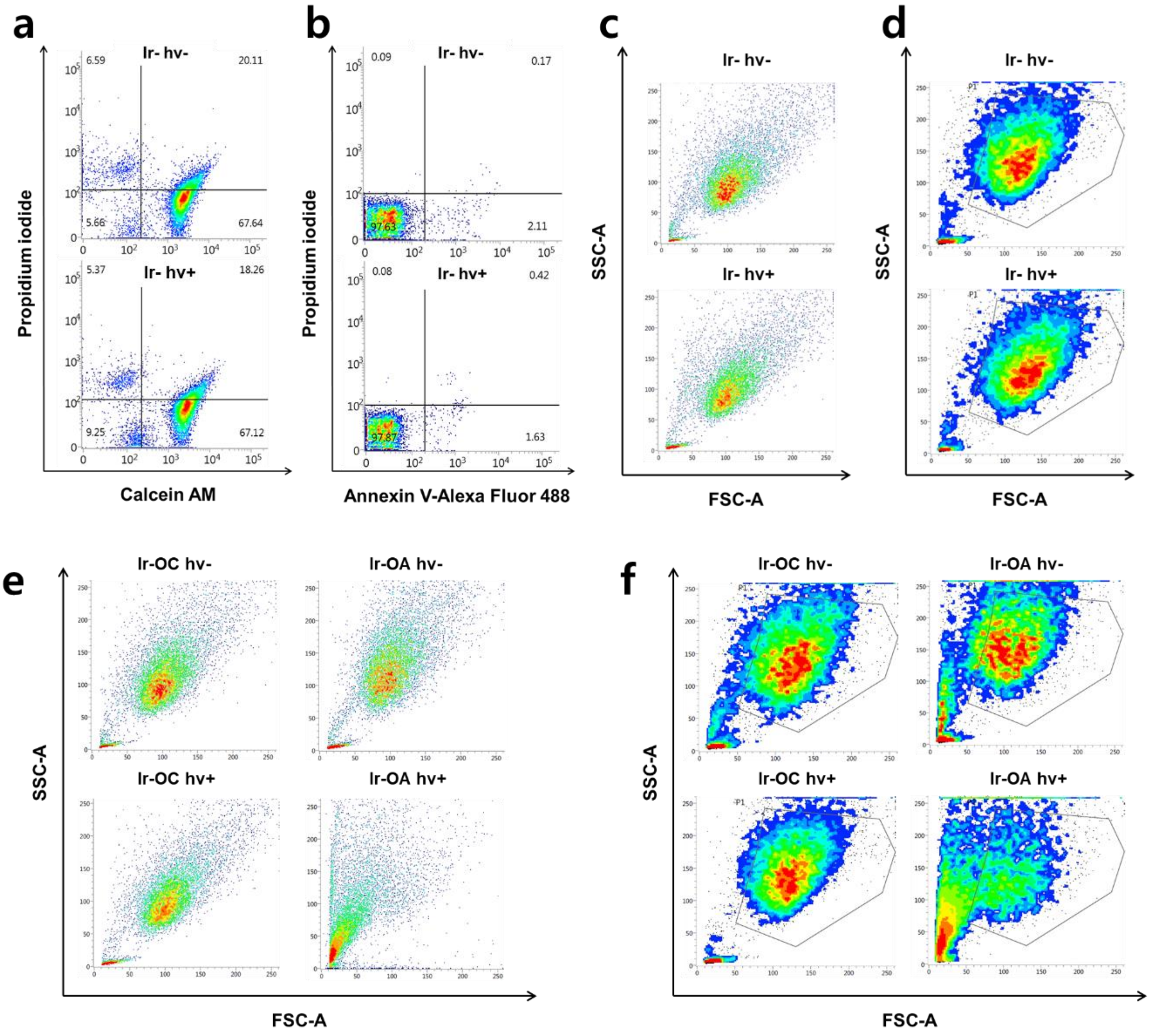

Supplementary Figure 15. Representative Calcein AM vs PI **(a)** and Annexin V vs PI **(b)** flow cytometry plot for HeLa cells without iridium complexes. Conditions: light source = 400 nm light LED array, light dose = 0.255 J cm^{-2}. **(c-f)** Gating strategies corresponding to flow cytometry plots of Supplementary Fig. 15a **(c)**, Supplementary Fig. 15b **(d)**, Fig. 3c **(e)** and Fig. 3d **(f)**. For the apoptosis assay (Annexin V/PI), the starting cells were selected by the preliminary FSC/SSC gates to find reasonable size cells with internal complexity. We choose cells with positive FSC and positive SSC, and discarded cells with extremely high SSC or FSC. The boundaries between positive/negative were 7 X 10^4, 7 X 10^4 (black line). For the Live/Dead assay (Calcein AM/PI), we allocated all cells into the experimental groups to count dead cells which could not maintain their internal complexity.

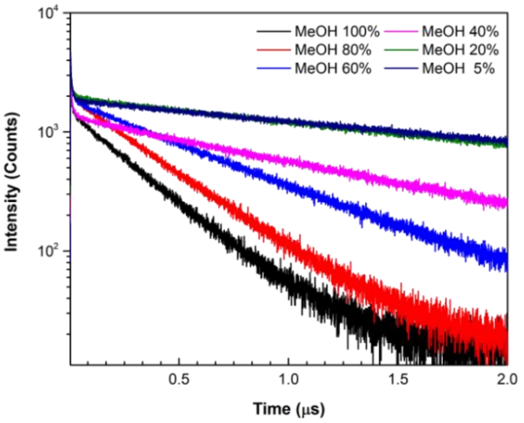

Supplementary Figure 16. Lifetime analysis for **Ir-OA** depending on viscosity. The viscosity of 5, 20, 40, 60, 80, and 100% (MeOH percent ratio in glycerol, v/v%) solution corresponds to 950, 250, 58, 13, 4.8, and 0.6 cP respectively. Conditions: [**Ir-OA**] = 20 μM. Source data are provided as a Source Data file.

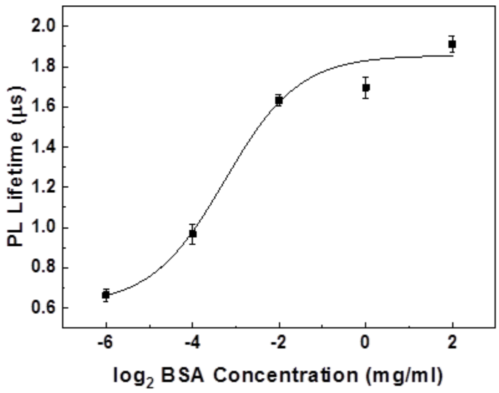

Supplementary Figure 17. The change in the lifetime of **Ir-OA** according to BSA concentration. The lifetime of **Ir-OA** was measured by TCSPC at each BSA concentration of 0.0156, 0.0625, 0.250, 1.000, and 4.000 mg/mL. conditions: [**Ir-OA**] = 20 μM, λ_{ex} = 450 nm. d., n=3. Data are presented as mean value ± s. d. Source data are provided as a Source Data file.

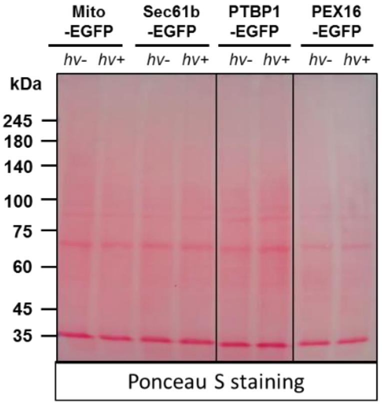

Supplementary Figure 18. Ponceau S staining for identifying protein loading quantity. The experiment was repeated three times independently, and each experiment showed similar results.

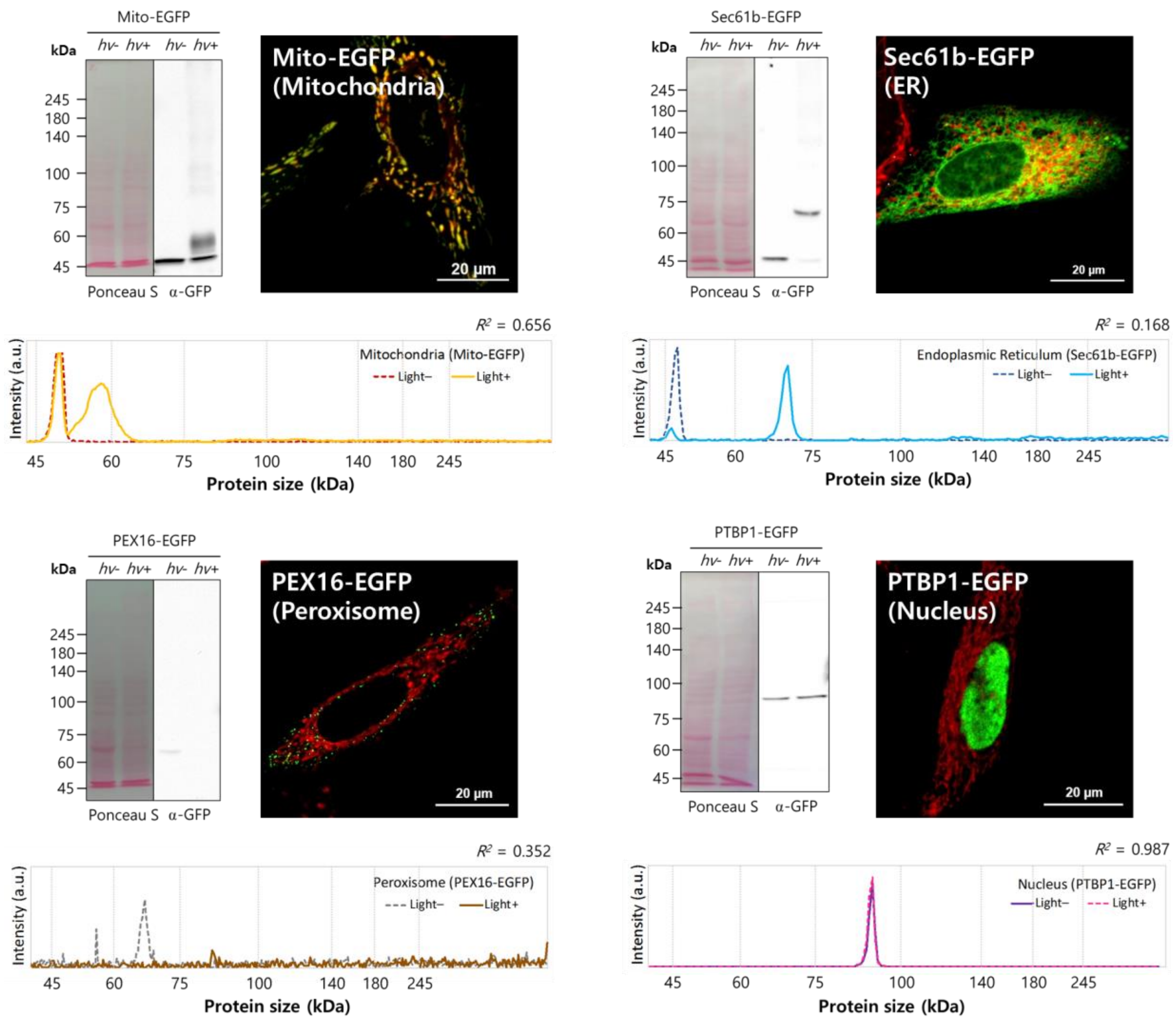

Supplementary Figure 19. Protein photo-crosslinking by photoactivation of **Ir-OA** in HeLa cells. Western blot (left) for identification of protein photo-crosslinking of **Ir-OA** in HeLa cells depending on four different cell organelles: mitochondria (Mito-EGFP), ER (Sec61b-EGFP), peroxisome (PEX16-EGFP), and nucleus (PTBP1-EGFP). Note that transfection with PEX16-EGFP construct in HeLa is insufficient, thereby, the signal on the lane after photo-crosslinking reaction does not seem to appear. Co-localization images (right) of **Ir-OA** (red signal) and each EGFP (green signal). l_{ex} = 405 nm and 488 nm (**Ir-OA** and EGFP, respectively). Pearson's coefficient for respective cell organelles with **Ir-OA** was calculated using Image J software (Mito-EGFP, $R = 0.891$; Sec61b-EGFP, $R = 0.634$; PEX16-EGFP, $R = 0.181$; PTBP1-EGFP, $R = 0.055$ vs **Ir-OA**). Imaging conditions: [**Ir-OA**] = 4 μM, λ_{ex} = 405 nm for **Ir-OA** , λ_{ex} = 488 nm for EGFP. Emission gain: 550–650 nm for **Ir-OA** and 500–550 nm for EGFP. Line-cut analysis (bottom) of Western blot signals with or without photo-irradiation was performed to quantify the crosslinking efficiency (η). Each correlation value (R^2) indicating similarity was written above the line cut spectrum. All imaging and blot experiments were repeated three times independently, and each experiment showed similar results. Source data are provided as a Source Data file.

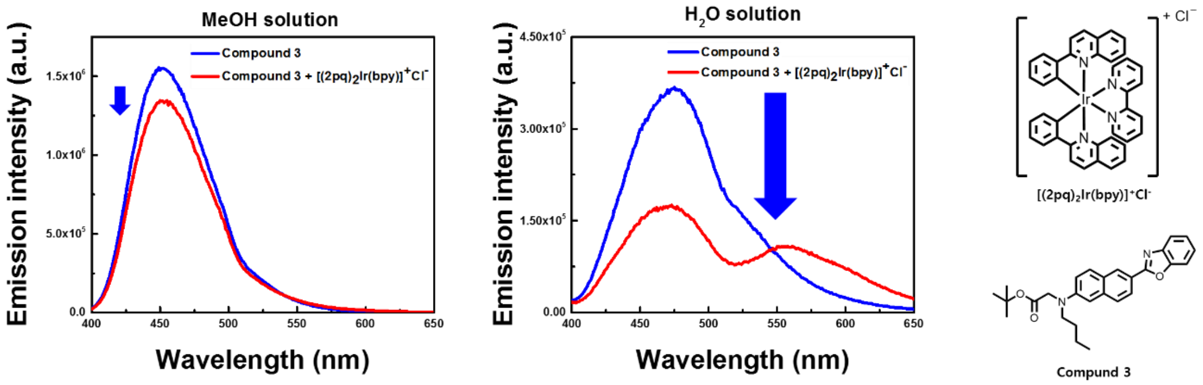

Supplementary Figure 20. Intermolecular energy transfer efficiency depending on solvent polarity. The photoluminescence spectrum of Compound 3 solution and Compound 3 + $[(2pq)_2Ir(bpy)]^+Cl^-$ solution in methanol or water. The decreased photoluminescence corresponds to intermolecular energy transfer efficiency from Compound 3 to $[(2pq)_2Ir(bpy)]^+Cl^-$. Conditions: [Compound 3] and $[[(2pq)_2Ir(bpy)]^+Cl^-]$ = 20 μM. Source data are provided as a Source Data file.

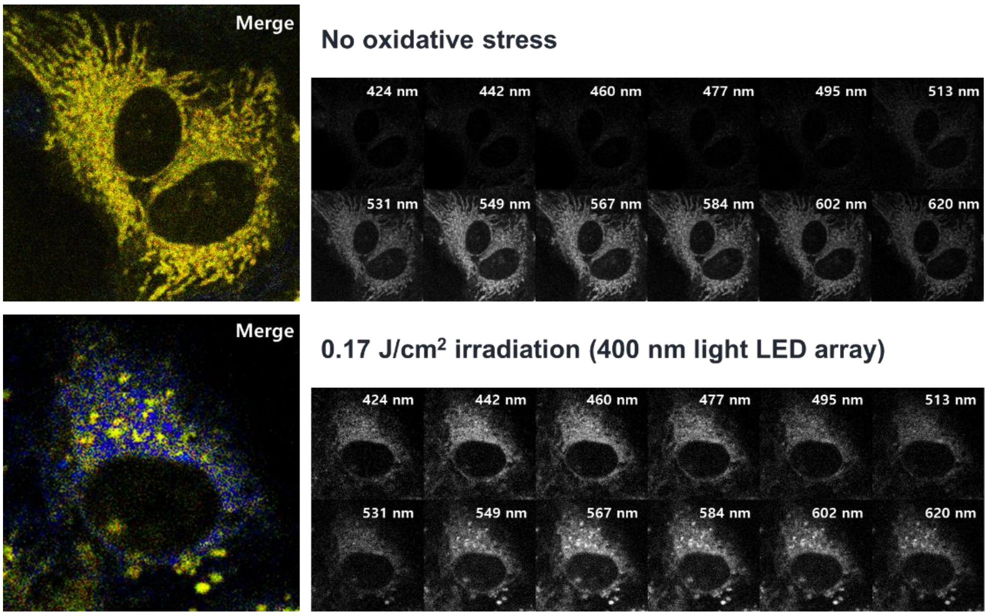

Supplementary Figure 21. Wavelength resolved CLSM images of mitochondrial depolarisation. Lambda scanning images was obtained from 424 nm to 620 nm at the 18 nm interval before/after irradiation. Merged images and their colour (left) describe overlapping images gained at each wavelength. Yellow region of merged image (left top) corresponds to polar mitochondria (there is no signal at blue region), and the blue region of merged image (left bottom) corresponds to depolarised mitochondria. Conditions: [**Ir-OA**] = 4 μM, light source = 400 nm light LED array (0.170 J cm^{-2}). The experiment was repeated three times independently, and each experiment showed similar results.

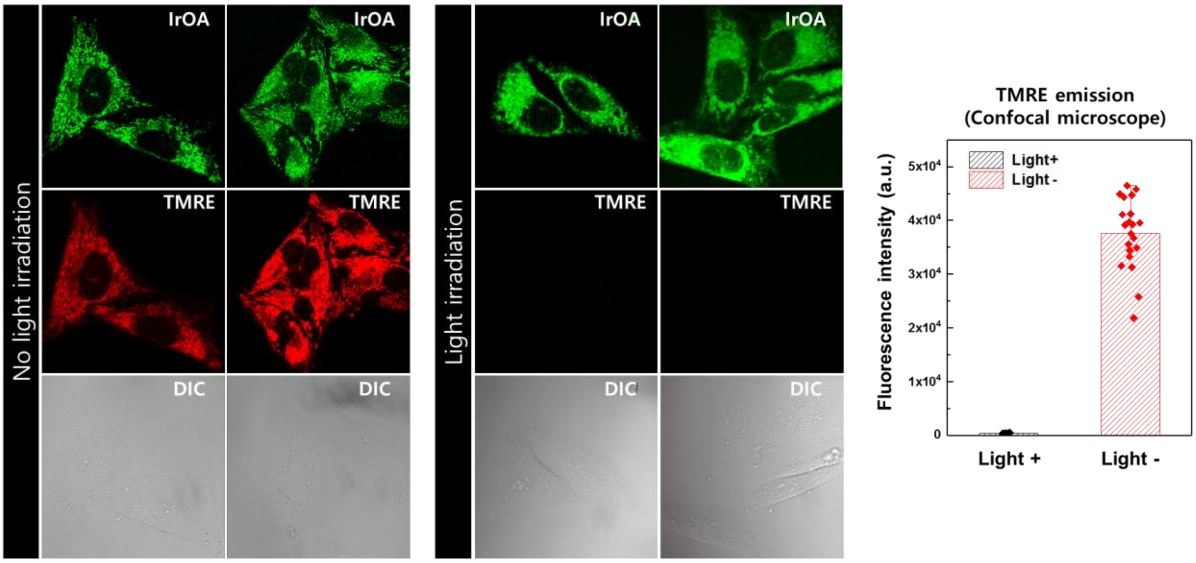

Supplementary Figure 22. TMRE assay for monitoring mitochondria depolarisation. The CLSM images for TMRE and **Ir-OA** 30 minutes after light irradiation (left), and averaged intensity of TMRE signal inside the cells (right). The averaged intensity was calculated from the randomly selected 21 cells (Light- condition) and 9 cells (Light+ condition). The selected regions are provided as a Source Data file. Conditions: [**Ir-OA**] = 4 μM. [TMRE] = 200 nM, light source = 400 nm light LED array, light dose = 0.170 J cm^{-2}. n=3 independent experiments were conducted. Data are presented as mean value ± s. d.

Supplementary Table 1. Photophysical properties for Compound 4, **Ir-OC**, and **Ir-OA**.

	λ_{abs} (nm), ε ($M^{-1}cm^{-1}$)	λ_{em} (nm)	Φ_{PL}
**Ir-OA**	346 (30,800), 389 (19,950), 438 (5,550)	470, 571	0.045
**Ir-OC**	339 (24,150), 438 (5,050)	561	0.089
Compound 4	364 (16,450), 389 (16,950)	482	0.929

The absorption and emission wavelengths and corresponding extinction coefficient (ε) were measured in the 20 μM water:DMSO = 99:1 (v/v) solutions of each sample. The Φ_{PL} values were calculated by the equation of $\Phi_{PL} = \Phi_{ref} \times (A_{ref}/A) \times (I/I_{ref})$ where A is absorbance, and I is integrated emission intensity (λ_{ex} = 400 nm)

Supplementary Table 2. Quantitative phototoxicity of **Ir-OA** according to irradiation energy.

	IC_{50}		
Irradiation energy	0.085 J/cm^2	0.170 J/cm^2	0.255 J/cm^2
MTT assay (n=4)	2.348 ± 0.162 μM	1.583 ± 0.003 μM	1.302 ± 0.052 μM
CCK-8 assay (n=4)	15.849 ± 0.370 μM	11.036 ± 0.610 μM	7.305 ± 0.758 μM

The IC_{50} values are measured by mitochondrial function-dependent (MTT assay) and independent (CCK-8) assay. The assays were triplicate at various irradiation energy (0.085, 0.170, and 0.255 J cm^{-2})

Supplementary Table 3. Detail information of prepared plasmids

Name (expected size)	Localization	Features	Promotor/ Vector	Details
Mito-EGFP (29.7 kDa)	Mitochondria	*NotI*-Mito-*BamHI*-EGFP-Stop-*XhoI*	CMV /pCDNA3	Mito-: MLATRVFSLVGKRAISTSVCVRAH (Matrix targeting sequence, Fornuskova et al., 2010)
PTBP1-EGFP (86.8 kDa)	Nucleus	*NotI*-PTBP1-*BamHI*-EGFP-Stop-*XhoI*	CMV /pCDNA3	PTBP1 (NM_002819)
Sec61b-mGFP (39.0 kDa)	Endoplasmic reticulum	*NheI-BglII*-Sec61b-*HindIII-EcoRI-SalI-SacII-ApaI-BamHI*-mGFP-Stop-*NotI*	CMV /pEGFP-N1	Sec61b (NM_006808)
PEX16-EGFP (65.8 kDa)	Peroxisome	*HindIII- KpnI*-PEX16-*BamHI-NheI*-EGFP-Stop-*NotI*	CMV /pCDNA3	PEX16 (NM_004813)

Supplementary References

1. Tanaka, K., Tainaka, K., Kamei, T.& Okamoto, A. Direct labeling of 5-methylcytosine and its applications. *J. Am. Chem. Soc.* **129,** 5612-5620 (2007).
2. Nam, J. S. et al. Endoplasmic reticulum-localized iridium(iii) complexes as efficient photodynamic therapy agents via protein modifications. *J. Am. Chem. Soc.* **138,** 10968-10977 (2016).
3. Suzuki, K. et al. Reevaluation of absolute luminescence quantum yields of standard solutions using a spectrometer with an integrating sphere and a back-thinned ccd detector. *Phys. Chem. Chem. Phys.* **11,** 9850-9860 (2009).
4. Lee, S. Y. et al. Apex fingerprinting reveals the subcellular localization of proteins of interest. *Cell. Rep.* **15,** 1837-1847 (2016).
