# Supplementary material for: Analysing the mechanism of mitochondrial oxidation-induced cell death using a multifunctional iridium(III) photosensitiser
Source: Nat Commun. 2021 Jan 4;12:26. doi: 10.1038/s41467-020-20210-3 (PMC7782791; doi:10.1038/s41467-020-20210-3)
Supplement: Supplementary file 3 — Descriptions of Additional Supplementary Files [file 41467_2020_20210_MOESM3_ESM.pdf]

Supplementary Data 1

Description: Proteomic analysis for methionine oxidised proteome. Analysis of the methionine-oxidised (O-Met) proteins in the whole cell proteome. The protein list represents $\log_2$(Fold change) and -logP values. The O-Met proteome was categorised into four main groups (channel, translocase, and OXPHOS complex; proteases; Mitochondrial fission and fusion related proteins; cytosol localised proteins). The source data have been deposited to the ProteomeXchange Consortium via the PRIDE partner repository with the dataset identifier PXD022163 [https://doi.org/10.6019/PXD022163].

Supplementary Movie 1

Description: Mitochondrial depolarisation with photoactivation of Ir-OA. Ratiometric CLSM imaging of HeLa cells with Ir-OA according to giving oxidative stress. The mitochondrial polarity change was monitored by the real-time ratiometric imaging during photoactivation within 110 s. The CLSM instrument's laser excited Ir-OA for real-time imaging. Ratio = (emission of acceptor, λ_{em} = 573–620 nm/emission of donor, λ_{em} = 420–480 nm). The green signal corresponds to normal mitochondria with high MMP.

Supplementary Movie 2

Description: Mitochondrial morphological change, representing mitochondrial matrix swelling, fission, and fusion. Time-lapse Airyscan 2 images of HeLa cells with Ir-OA during oxidative stress exposure (14 mW, 405 nm laser of laser scanning microscopy) for 323 s. To monitor morphological changes, the mitochondrial matrix was transfected by Mito-EGFP (green signal).

Supplementary Movie 3

Description: Magnified movie to show mitochondrial fission. Enlarged time lapse imaging (0–340 s) of white boxes from Fig. 7a. Along with mitochondrial fission, matrix swelling was also observed.

Supplementary Movie 4

Description: Magnified movie to show mitochondrial fusion. Enlarged time lapse images (0–340 s) of white boxes from Fig. 7a. Along with mitochondrial fusion, matrix swelling was also observed.
