# Supplementary material for: Analysing the mechanism of mitochondrial oxidation-induced cell death using a multifunctional iridium(III) photosensitiser
Source: Nat Commun. 2021 Jan 4;12:26. doi: 10.1038/s41467-020-20210-3 (PMC7782791; doi:10.1038/s41467-020-20210-3)
Supplement: Supplementary file 9 — Reporting Summary [file 41467_2020_20210_MOESM9_ESM.pdf]

nature research

Corresponding author(s): Tae-Hyuk Kwon

Last updated by author(s): Oct 21, 2020

Reporting Summary

Nature Research wishes to improve the reproducibility of the work that we publish. This form provides structure for consistency and transparency in reporting. For further information on Nature Research policies, see our Editorial Policies and the Editorial Policy Checklist.

Statistics

For all statistical analyses, confirm that the following items are present in the figure legend, table legend, main text, or Methods section.

n/a	Confirmed	
☐	☒	The exact sample size (*n*) for each experimental group/condition, given as a discrete number and unit of measurement
☐	☒	A statement on whether measurements were taken from distinct samples or whether the same sample was measured repeatedly
☐	☒	The statistical test(s) used AND whether they are one- or two-sided<br>*Only common tests should be described solely by name; describe more complex techniques in the Methods section.*
☒	☐	A description of all covariates tested
☒	☐	A description of any assumptions or corrections, such as tests of normality and adjustment for multiple comparisons
☐	☒	A full description of the statistical parameters including central tendency (e.g. means) or other basic estimates (e.g. regression coefficient) AND variation (e.g. standard deviation) or associated estimates of uncertainty (e.g. confidence intervals)
☐	☒	For null hypothesis testing, the test statistic (e.g. *F*, *t*, *r*) with confidence intervals, effect sizes, degrees of freedom and *P* value noted<br>*Give P values as exact values whenever suitable.*
☒	☐	For Bayesian analysis, information on the choice of priors and Markov chain Monte Carlo settings
☒	☐	For hierarchical and complex designs, identification of the appropriate level for tests and full reporting of outcomes
☐	☒	Estimates of effect sizes (e.g. Cohen's *d*, Pearson's *r*), indicating how they were calculated

Our web collection on statistics for biologists contains articles on many of the points above.

Software and code

Policy information about availability of computer code

Data collection	ZEN software (ver. 3.0) from Carl Zeiss was utilized for collection of confocal imaging (localization and mitochondria dynamics).<br>LC-MS/MS data acquisition software Xcalibur (ver. 4.1.31.9) from Thermofisher Scientific was utilized.
Data analysis	Microscopy Imaging analyses used Carl Zeiss ZEN 3.0 blue edition.<br>Sequest Sorcerer platform and Uniprot Homo sapiens protein sequence database (42284 entries) were utilized for MS/MS analysis.<br>vr. 4.9.0 Scaffold from Proteome Software Inc. was used to validate MS/MS-based peptide and protein identification (PyMOL ver1.7.4.4).<br>Image J (ver. 1.52p) and MetaMorph software (ver. 7) was also utilized for colocalization and crosslinking correlation analysis.<br>OriginPro 2017, Microsoft office 2017 were also used.<br>In PLIM images, exponential fitting for the obtained fluorescence decays was accomplished using the Symphotime-64 software (Ver. 2.2).<br>The FACS data was processed by the BD FACSuite software (ver. 1.0.6)

For manuscripts utilizing custom algorithms or software that are central to the research but not yet described in published literature, software must be made available to editors and reviewers. We strongly encourage code deposition in a community repository (e.g. GitHub). See the Nature Research guidelines for submitting code & software for further information.

Data

Policy information about availability of data

All manuscripts must include a data availability statement. This statement should provide the following information, where applicable:

- Accession codes, unique identifiers, or web links for publicly available datasets
- A list of figures that have associated raw data
- A description of any restrictions on data availability

The authors declare that the data supporting the findings of this study are available within the article and its Supplementary Information. The crystal structures of

proteins were visualised and processed with PyMOL (https://pymol.org/2/), and the protein information utilised in this study is available from the RCSB protein data bank (PBD ID) (http://www.rcsb.org/pdb/) and the Homo sapiens protein sequence database (42284 entries, UniProt (http://www.uniprot.org/)). The mass spectrometry proteomics data that support the findings of this study have been deposited to the ProteomeXchange Consortium via the PRIDE partner repository with the dataset identifier PXD022163 and 10.6019/PXD022163. Extra data are available from the corresponding author upon reasonable request. The source data underlying Figs. 1c-f, 2b-c, 3a, 4a-f, and 5b, and Supplementary Figs. 13, 17, 19, 20, and 22 are provided as a Source Data file.

Field-specific reporting

Please select the one below that is the best fit for your research. If you are not sure, read the appropriate sections before making your selection.

[x] Life sciences [ ] Behavioural & social sciences [ ] Ecological, evolutionary & environmental sciences

For a reference copy of the document with all sections, see nature.com/documents/nr-reporting-summary-flat.pdf

Life sciences study design

All studies must disclose on these points even when the disclosure is negative.

Sample size	To the imaging analyses, cell viability test, and the other assays, we followed sample size which general research papers published (Nam et al. J. Am. Chem. Soc. 2016, 138, 34, 10968–10977). To the proteomic analyses, the experiments were triplicated with four conditions (Ir- hv-/Ir- hv+/Ir+ hv-/Ir+ hv+). In sample preparation, total 50 µg whole cell proteins were used for in-gel digestion.
Data exclusions	No data were excluded from the analyses
Replication	All reported results were triplicated independently. Each attempts showed similar results.
Randomization	For the experiments using cell line (O-Met proteomics, imaging analysis/ cell viability test), we randomly allocated cells cultured in the same condition into experimental sets including positive/negative control. In imaging analysis, we randomly selected target at least three individual cells, then we choosed the best images.
Blinding	In the process of allocating experimental groups, the investigators were totally blinded all about experiment-related factors.

Reporting for specific materials, systems and methods

We require information from authors about some types of materials, experimental systems and methods used in many studies. Here, indicate whether each material, system or method listed is relevant to your study. If you are not sure if a list item applies to your research, read the appropriate section before selecting a response.

Materials & experimental systems

n/a	Involved in the study	
[ ]	[x]	Antibodies
[ ]	[x]	Eukaryotic cell lines
[x]	[ ]	Palaeontology and archaeology
[x]	[ ]	Animals and other organisms
[x]	[ ]	Human research participants
[x]	[ ]	Clinical data
[x]	[ ]	Dual use research of concern

Methods

n/a	Involved in the study	
[x]	[ ]	ChIP-seq
[ ]	[x]	Flow cytometry
[x]	[ ]	MRI-based neuroimaging

Antibodies

Antibodies used	GFP monoclonal antibody, applicable in Figure 4, Thermofisher Scientific, MA5-15256, GFP28R; Goat anti-Mouse IgG (H+L) Secondary Antibody, HRP; applicable in Figure 4, Thermofisher Scientific, 31430
Validation	All antibodies were purchased from Thermofisher Scientific. The manufacturer provides its application on their website (https://www.thermofisher.com/antibody/product/Goat-anti-Mouse-IgG-H-L-Secondary-Antibody-Polyclonal/31430 and https://www.thermofisher.com/antibody/product/GFP-Monoclonal-Antibody-GF28R/MA5-15256).<br><br>Additionally, western blot signals for each four different eGFP constructs were displayed at the desired molecular weight, and the secondary antibody (Goat anti-Mouse IgG (H+L)) was validated by Western blot detection for eGFP in Figure 4.

Eukaryotic cell lines

Policy information about cell lines

Cell line source(s)	HeLa: Korean Cell Line Bank (KCLB) and HEK293T:ATCC
Authentication	The cells were not authenticated before use
Mycoplasma contamination	The cell lines were tested negative for mycoplasma contamination
Commonly misidentified lines (See ICLAC register)	None of these cell lines was used

Flow Cytometry

Plots

Confirm that:

- [x] The axis labels state the marker and fluorochrome used (e.g. CD4-FITC).
- [x] The axis scales are clearly visible. Include numbers along axes only for bottom left plot of group (a 'group' is an analysis of identical markers).
- [x] All plots are contour plots with outliers or pseudocolor plots.
- [x] A numerical value for number of cells or percentage (with statistics) is provided.

Methodology

Sample preparation	HeLa cells (Korean Cell Line Bank, KCLB) were prepared on the 6-well plate with culture media (DMEM (Gibco) supplemented with 10% FBS, 50 units/mL penicillin, and 50 ug/mL streptomycin). The cells were incubated with Ir-OA and Ir-OC, then irradiated by a blue LED array (λ = 400 nm, 255 mJ/cm2). After several hours, the adherent cells were harvested and washed with cold PBS, followed by suspending in PBS containing dyes for Annexin V/PI staining and Calcein AM/PI staining. Further information is provided in the supporting information.
Instrument	BD FACSVerseTM (BD bioscience, US)
Software	BD FACSuite v1.0.6
Cell population abundance	The abundance of the HeLa cell population within post-sort fractions is 10000. We selected experimental cells of reasonable size (positive FSC) with internal complexity (positive SSC) except for cell debris . The purity of the samples is above 80%
Gating strategy	The starting cells were selected by the preliminary FSC/SSC gates to find reasonable size cells with internal complexity. We choose cells with positive FSC and positive SSC, and discarded cells with extremely high SSC or FSC. The boundaries between positive/negative were 7 X 10^4, 7 X 10^4 (Annexin V/PI) respectively. For the Live/Dead assay (Calcein AM/PI), We allocated all cells into the experimental groups because we had to count dead cells which could not maintain their internal complexity. Even though little amount of cell debris can be included to the experiments, we confirmed significant different results between control groups and experimental group.

- [x] Tick this box to confirm that a figure exemplifying the gating strategy is provided in the Supplementary Information.
